# Striatal Neuropeptides Enhance Selection and Rejection of Sequential Actions

**DOI:** 10.3389/fncom.2017.00062

**Published:** 2017-07-27

**Authors:** David Buxton, Enrico Bracci, Paul G. Overton, Kevin Gurney

**Affiliations:** Adaptive Behaviour Research Group, Department of Psychology, The University of Sheffield Sheffield, United Kingdom

**Keywords:** action selection, action sequence, basal ganglia, chunking, enkephalin, neuropeptides, striatum, substance P

## Abstract

The striatum is the primary input nucleus for the basal ganglia, and receives glutamatergic afferents from the cortex. Under the hypothesis that basal ganglia perform action selection, these cortical afferents encode potential “action requests.” Previous studies have suggested the striatum may utilize a mutually inhibitory network of medium spiny neurons (MSNs) to filter these requests so that only those of high salience are selected. However, the mechanisms enabling the striatum to perform clean, rapid switching between distinct actions that form part of a learned action sequence are still poorly understood. Substance P (SP) and enkephalin are neuropeptides co-released with GABA in MSNs preferentially expressing D_1_ or D_2_ dopamine receptors respectively. SP has a facilitatory effect on subsequent glutamatergic inputs to target MSNs, while enkephalin has an inhibitory effect. Blocking the action of SP in the striatum is also known to affect behavioral transitions. We constructed phenomenological models of the effects of SP and enkephalin, and integrated these into a hybrid model of basal ganglia comprising a spiking striatal microcircuit and rate–coded populations representing other major structures. We demonstrated that diffuse neuropeptide connectivity enhanced the selection of unordered action requests, and that for true action sequences, where action semantics define a fixed structure, a patterning of the SP connectivity reflecting this ordering enhanced selection of actions presented in the correct sequential order and suppressed incorrect ordering. We also showed that selective pruning of SP connections allowed context–sensitive inhibition of specific undesirable requests that otherwise interfered with selection of an action group. Our model suggests that the interaction of SP and enkephalin enhances the contrast between selection and rejection of action requests, and that patterned SP connectivity in the striatum allows the “chunking” of actions and improves selection of sequences. Efficient execution of action sequences may therefore result from a combination of ordered cortical inputs and patterned neuropeptide connectivity within striatum.

## 1. Introduction

The problem of action selection is common to all animal life: how best to utilize the body's limited resources when faced with almost limitless behavioral possibilities in a complex and changing environment? A mechanism for deciding on the optimal action in every situation is necessary, and a growing consensus supports the hypothesis that the basal ganglia are the neural structures primarily responsible for action selection in vertebrates (Doya, 1999; Redgrave et al., 1999; Prescott, 2007). Under this hypothesis the cortex, thalamus and other afferent regions generate “requests for action” that are filtered by the basal ganglia before being granted access to the motor plant, ensuring that only one request for any muscle group or body part is acted upon at a time and avoiding problematic conflicts.

The striatum is the primary input nucleus to the basal ganglia. Its neuronal population consists of at least 90% GABAergic medium spiny neurons (MSNs) (Rymar et al., 2004) with the remainder one of several types of interneuron. The striatum has a broadly homogeneous appearance and no clear structural organization (Kreitzer, 2009), though histological staining reveals a somatotopic layout in regions receiving sensorimotor afferents (Yelnik, 2002; Nambu, 2011). This topographic organization extends throughout the basal ganglia, with multiple parallel loops processing sensorimotor, associative, and limbic information preserved in all basal ganglia structures (Nakano et al., 2000; Yelnik, 2002; McHaffie et al., 2005), suggesting that the selection process is not limited to motor functions but applies to a wide array of cognitive and motivational domains.

MSNs can be subdivided into one of two types based on the preferential expression of dopamine receptor subtypes and their projection targets; D_1_ (or striatonigral) MSNs preferentially express D_1_ dopamine receptors and project to internal regions of the globus pallidus and the SNr, and D_2_ (or striatopallidal) MSNs preferentially express D_2_ dopamine receptors and project to external regions of globus pallidus (Smith et al., 1998). These two pathways from cortical input to basal ganglia output have classically been referred to as “direct” and “indirect” respectively, though their roles in the process of action selection have been the subject of much debate (Donahue and Kreitzer, 2015; O'Hare et al., 2016; Yin, 2016).

The specifics of the striatum's function are not fully known, but the dense network of inhibitory intraconnections and the somatotopic organization of the structure allows for a broad hypothesis. Under the action selection hypothesis, cortical regions encoding complementary aspects of a “request for action” project to an MSN population that competes via mutual inhibition with separate populations receiving inputs encoding alternate requests. The common currency of input salience allows afferents from various cortical regions representing a range of contextual information to compete across domains, and this competition between multiple action requests allows the MSN population receiving the inputs of greatest salience to disinhibit their downstream targets and permit the execution of that action request.

The concatenation of individual actions into sequences is a trait seen in many different species, and the smooth execution of learned action sequences is fundamental to behaviors such as singing in birds, grooming in rats, and everyday activities such as tying shoelaces or buttoning a shirt in humans. Once initiated, action sequences can be executed smoothly and with an almost unconscious automaticity that allows other unrelated actions to be carried out simultaneously (Seger and Spiering, 2011). This “chunking” of multiple actions into a single unit is cognitively efficient (Ramkumar et al., 2016) and makes action sequences highly valuable; action sequences are part of so many routine behaviors that their impairment in conditions such as Huntington's disease or Parkinson's disease (Benecke et al., 1987; Agostino et al., 1992) is one of the primary symptoms of these conditions.

Sequences have importance beyond improving the efficiency of everyday motor actions. Sequences exist in a hierarchical organization that enables both goal–directed and habitual behavior (Dezfouli and Balleine, 2013), and this layered control allows for a wide range of higher cognitive processes (Graybiel and Grafton, 2015; Savalia et al., 2016). However, despite the crucial importance of action sequences there is currently limited understanding of their neurological basis; it is not clear what exactly is coded by cortical afferents to striatum, the level of description encapsulated by an action request, which aspects of sequence learning occur in the cortex or basal ganglia, or the neurological changes that underlie sequence learning. It is also unclear what allows for the rapid, smooth transitions between distinct actions, or how multiple actions are chunked together into a learned sequence. The striatum has been strongly implicated in the formation, concatenation, and execution of action chunks (Graybiel, 1998; Jog et al., 1999; Wymbs et al., 2012; Jin et al., 2014), so recent neurophysiological studies of neuropeptides in the striatum may provide insight into some of these problems.

Substance P (SP) and enkephalin are neuropeptides co-released with GABA from D_1_ or D_2_ MSNs respectively. These neuropeptides have been shown to have an effect on subsequent glutamatergic inputs to the MSNs they target; SP is facilitatory, acting on presynaptic NK_1_ receptors and causing up to an average 47% increase in glutamatergic EPSPs on timescales from 100 ms to ~1 s (Blomeley and Bracci, 2008). Enkephalin is inhibitory, acting on μ- and δ-opioid receptors and causing up to an average 30% reduction of glutamatergic EPSPs on timescales from 500 ms to ~2s (Blomeley and Bracci, 2011). The function of these neuropeptides in the context of the striatum and action selection has so far been unclear, though the effect of SP in particular is of interest as it strongly implies a co-operative role for what has previously been thought to be a purely competitive inhibitory network. Notably, blocking the action of SP has been shown to decrease amphetamine-induced behavior in rats (Gonzalez-Nicolini and McGinty, 2002) and mice lacking NK_1_ receptors exhibit behavioral traits comparable to ADHD (Yan et al., 2009; Porter et al., 2015).

We propose that the action and interaction of these neuropeptides plays a crucial role in enabling corticostriatal networks to encode action sequences, and that their combined effects allow successive actions in a sequence to be selected and executed with greater efficiency than a comparable non-sequence action. This would assist with rapid, smooth transitions between sequential actions while still allowing for sequences to be interrupted by the arrival of an exceptionally salient action request. We present a hybrid computational model of the basal ganglia consisting of both spiking and rate–coded components that tests this hypothesis using several different neuropeptide connectivity configurations.

## 2. Materials and methods

The hybrid model consists of two primary units:

1. A spiking striatal microcircuit model comprising MSNs, fast-spiking interneurons (FSIs), and associated neurotransmitters and neuropeptides
2. A rate–coded model of other basal ganglia structures, ventrolateral thalamus, and motor cortex

Embedding the striatal microcircuit model in a model of the basal ganglia–thalamocortical loop allows the results and implications of striatal computation to be explored at the level of motor cortex output, providing a closer correspondence to behavior than analysis of MSN activity. An overview of each model will be followed by an additional section detailing the metrics used to define selection. A full technical description of both models following the format proposed by Nordlie et al. (2009) can be found in the Supplementary Material.

### 2.1. Hybrid model construction

The striatal microcircuit model closely follows the descriptions in Humphries et al. (2009a,b), with updates to some parameters as described in Tomkins et al. (2014). An outline of the model following the structure in Table S1 will be given here but for greater explanatory detail we refer the reader to these sources. Unless explicitly stated otherwise all models described here were instantiated in the SpineCreator environment (Cope et al., 2017) and executed using the BRAHMS simulation engine (Mitchinson et al., 2010) with a forward Euler solver at a 0.1 ms timestep.

The basal ganglia–thalamocortical loop (or BG loop) model is mostly unchanged from the thalamocortical loop (TC) model described in Humphries and Gurney (2002). An overview of the model following the structure in Table S11 will be given here but for greater explanatory detail we refer the reader to that paper. The integration of the spiking striatal microcircuit with the rate–coded basal ganglia model is novel to the hybrid model and will be described in more detail.

#### 2.1.1. Neuron populations

The striatal microcircuit model is composed of 6,000 spiking MSNs divided into two equal groups of 3,000 D_1_ and D_2_–type MSNs. These are further subdivided into six “action channels” *c*_1_…*c*_6_ of 500 neurons each, representing the striatal targets of six distinct cortical action requests (Table S2). The MSN population is complemented by an additional 60 FSIs — 1% of the MSN population (Luk and Sadikot, 2001; Humphries et al., 2010) — that receive equal innervation from each action request.

The BG loop model is composed of five populations representing major structures within the basal ganglia–thalamocortical loop. Each population is composed of six separate leaky integrators representing a distinct “action channel” *c*_1_…*c*_6_ as in the striatal microcircuit model (Table S12).

#### 2.1.2. Connectivity

For MSN → MSN, FSI → MSN, and FSI → FSI projections within the striatal microcircuit model, an exhaustive all–to–all list of connections between the two populations is probabilistically culled according to the expected number of afferent connections for each type (Table S3). Culling is entirely independent of the action channel represented by a given neuron. In this manner, each neuron receives approximately the correct number of inputs of each type, but the overall model lacks any topology and is similar to the *random* model from Tomkins et al. (2014). All 500 D_1_ or D_2_ MSNs in channel *c*_*n*_ also project to the single neuron representing channel *c*_*n*_ in GPi/SNr or GPe respectively via a spike–to–rate conversion.

Neuropeptide projections co-exist with MSN → MSN GABA connections targeting both D_1_ and D_2_ MSNs (Yung et al., 1996; Blomeley et al., 2009; Blomeley and Bracci, 2011) and never appear on their own; however, GABA connections without an associated neuropeptide projection are permitted. A distinction is also made between three neuropeptide projection configurations, described in Section 2.2.

With the exception of STN, all BG loop populations are connected with one–to–one links to preserve the channel–based architecture of the basal ganglia (Table S13). The combination of diffuse excitatory STN and focused inhibitory striatal projections to GPe and GPi/SNr models an off–center, on–surround pattern of activation (Mink and Thach, 1993; Nambu et al., 2002) that selectively disinhibits the MCtx action channel corresponding to the selected action request. This is consistent with research showing that SNr neurons gate the flow of information to motor output regions and that their inhibition predicts motor activity (Deniau et al., 2007; Freeze et al., 2013).

#### 2.1.3. Neuron and synapse models

The striatal microcircuit model closely follows the descriptions in Humphries et al. (2009a,b), with updates to some parameters as described in Tomkins et al. (2014). The spiking MSN uses the canonical model of Izhikevich (2007) with updates from Humphries et al. (2009a) that capture the effects of dopamine modulation, and we reuse without modification the dopamine–modulated FSI model of Humphries et al. (2009b). Full descriptions of neuron models used in the striatal microcircuit are in Table S4.

The synaptic models of GABA and glutamate from Humphries et al. (2009b) are modified with the inclusion of a saturation effect from Tomkins et al. (2012), though for non-NMDA currents the saturation value is set high enough to have negligible effects in normal conditions. The glutamate models are further modified with the inclusion of phenomenological models of the effects of SP and enkephalin to provide a final input to striatal MSNs (Table S5).

Neural dynamics and connectivity for the BG loop model are unchanged from Humphries and Gurney (2002), with a few notable exceptions:

1. The connection weight *w*_sc–mc_ from sensory cortex to motor cortex is reduced to 0.5 from 1 in order to emphasize the role of GPi/SNr in disinhibiting VLT and thereby promoting the selected action request.
2. The connection weight *w*_vlt–mc_ from VLT to motor cortex is increased to 1.05 from 1 in order to allow for a stable MCtx–VLT feedback loop while GPi/SNr output remains below 0.05 (the GPi/SNr threshold in Humphries and Gurney, 2002).
3. Leaky integrator populations representing striatum are not used beyond model calibration and are replaced with the striatal microcircuit model.

Full details of leaky integrator dynamics and properties for the BG loop model are in Tables S14, S16.

#### 2.1.4. Neuropeptide models

We used available neurophysiological data to create phenomenological models of SP and enkephalin's effects on glutamatergic afferents to MSNs. Substance P is known to have both direct (Blomeley and Bracci, 2008) and indirect (Blomeley et al., 2009) effects on target MSNs; only the indirect effects are modeled here as it is unlikely that neuropeptides mediate direct communication between MSNs (Blomeley et al., 2009).

Neuropeptide action is simulated in two stages. The amount of neuropeptide released in response to a given level of MSN activity is calculated using a simple sum of exponentials, and this value is then converted into a facilitation or inhibition effect multiplier by means of a tuned response curve.

Thus, for a single spike-induced neuropeptide release event at time *t*_*i*_ the amplitude $a_{p}^{i}\left(t\right)$ of neuropeptide *p* induced by this event is given by:

(1) $$a_{p}^{i}(t)=S_{p}\left[\exp\left(\frac{-(t-t_{i})}{\tau_{f}^{p}}\right)-\exp\left(\frac{-(t-t_{i})}{\tau_{r}^{p}}\right)\right]$$

where *p* is either SP or enkephalin. τ_*r*_ and τ_*f*_ represent rise and fall time constants respectively, and *S*_*p*_ is the number of incoming spikes causing release of neuropeptide *p*. Multiple events over a period of time combine to form a net amplitude:

(2) $$\begin{array}{c} A_{p}(t)=\underset{i}{\sum^{\text{​}}}a_{p}^{i}(t) \end{array}$$

The net amplitude *A*_*p*_(*t*) of neuropeptide release determines the resulting modulatory effect *N*_*p*_(*t*), which is normalized using the Weibull cumulative distribution function and an additional gain factor β:

(3) $$N_{p}(t)=\beta_{p}\left[1-\exp{\left(-\frac{A_{p}(t)}{\lambda_{p}}\right)}^{b_{p}}\right]$$

This effect is appended to the synaptic input equation giving a final form for glutamate input to MSNs:

(4) $$I_{z}^{\text{ms}}={\bar{g}}_{z}h_{z}(E_{z}-v)\left[1+N_{\text{sp}}(t-\tau_{d}^{\text{sp}})\right]\left[1-N_{\text{enk}}(t-\tau_{d}^{\text{enk}})\right]$$

where *z* is either AMPA or NMDA. As the model is purely phenomenological, the delay between MSN activity and the onset of neuropeptide effects (Blomeley et al., 2009; Blomeley and Bracci, 2011) is captured using a fixed time offset τ_*d*_.

#### 2.1.5. Inputs

Both the striatal microcircuit model and the BG loop model receive external input from populations of Poisson spike generators representing action requests from sensory cortex. (We refer to inputs as originating from sensory cortex so that the model may represent a complete sensorimotor loop; however, inputs are entirely abstracted and could similarly originate from any non-motor cortical source that provides the striatum with patterned inputs.) Each spike source population comprises 500 separate Poisson spike generators, collectively defined as representing sensory cortex activity corresponding to a single action request. Each spike generator ${\text{sc}}_{c}^{i},1\leq i\leq 500$ has a one–to–one connection with a single D_1_ MSN and a single D_2_ MSN, and each motor cortex neuron mc_*c*_ projects to D_1_ and D_2_ MSNs ${\text{ms}}_{c}^{i},1\leq i\leq 500$ (Table S6). In contrast, the first 60 spike generators in each channel have a one–to–one connection to a single FSI and each motor cortex neuron projects to all 60 FSIs. Each striatal neuron therefore receives the same number of afferent connections from sensory cortex as from motor cortex. All 500 spike generators in each action channel also project to the single neuron representing that channel in STN and MCtx within the BG loop model (Table S15).

All six action channels are therefore uniquely represented in the striatal model by a distinct population of 1,000 MSNs split evenly into D_1_ and D_2_ subtypes, while each FSI receives input from all channels. This corticostriatal connectivity reflects the convergence of cortical afferents from functionally related cortical regions on target MSNs (Flaherty and Graybiel, 1993; Takada et al., 1998) and widespread input to target FSIs (Ramanathan et al., 2002; Berke, 2008) that provide distributed inhibition of MSNs. This represents an on–centre, off–surround pattern of corticostriatal connectivity that could support selection via activation of specific MSN populations.

#### 2.1.6. Integration of model components

The integration of rate–coded and spiking populations into a single model necessitates the creation of neural interconnects to translate activity rate to spiking output and vice–versa.

##### Rate–to–spike conversion

Activity rate output from motor cortex in the basal ganglia–thalamocortical loop model is converted to spike trains and projected to each MSN and FSI in the striatal model. Conversion is achieved by assigning a Poisson spike generator and a random number generator *P* to every striatal projection from motor cortex, and generating a spike every timestep on any connection where the activity rate *y*_mc_ is greater than *P* up to a maximum possible firing rate *r*_max_. The rate–to–spike conversion is thus achieved by:

(5) $$\text{Emit spike if }y_{\text{mc}}\;r_{\text{max}}\;\tau_{\text{bg}}>P$$

where τ_bg_ is the timestep value for the overall simulation.

##### Spike–to–rate conversion

Converting ongoing spike train activity into a normalized activity rate is necessary for the connections between model input and motor cortex, and for projections from striatal MSNs to GPe and GPi/SNr. An instantaneous measurement of spiking output is insufficient to generate a continuous activity rate, so a sum of exponentials captures a dynamic rate *r* of sensory cortex or MSN spiking which is then converted to an activity rate *y* in the range 0–1. The running mean of spiking activity is thus captured by:

(6) $$r_{s}(t)=\underset{i}{\sum^{\text{​}}}S_{s}\left[\exp\left(\frac{-(t-t_{i})}{\tau_{f}}\right)-\exp\left(\frac{-(t-t_{i})}{\tau_{r}}\right)\right]$$

where *s* is sensory cortex, D_1_ MSN, or D_2_ MSN and *S*_*s*_ is the number of spikes arriving from population *s*. The activity rate *y* is then given by:

(7) $$y_{s}(t)=1-\exp{\left(-\frac{r_{s}(t)}{\lambda_{s}}\right)}^{b_{s}}$$

**Figure 1:**
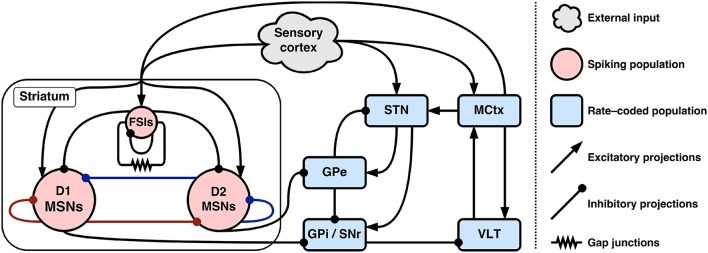
Connectivity of the hybrid basal ganglia model. Colored inhibitory connections in striatum indicate co-release of either substance P (red) or enkephalin (blue) with GABA.

Figure 1 gives an overview of the entire hybrid model and baseline connectivity between populations.

### 2.2. Action groups and selection metrics

In order to explore the effects of striatal neuropeptides on selection within the basal ganglia, several different action selection scenarios are simulated. To maintain consistency with Humphries and Gurney (2002) all simulations are conducted with a six–channel model. Action channels *c*_1_…*c*_4_ represent specific actions within an action group, and channel *c*_5_ represents a generic action that marks the end of every action group. Channel *c*_6_ is a null channel that receives no external input, except for in Section 3.4 where it is utilized as an intrusive “distractor” action.

In assessing model performance, a distinction is made between three types of action groups. The term *action series* refers to any group of action requests that occur one after the other but have no preferred semantic order; for example, taking a sip of tea; putting on glasses; scratching the nose. The specific order in which these actions occur is not important, but it is important that selection of more than one does not occur simultaneously. For the purposes of what follows, the four actions 1…4 in any order comprise a valid *action series*.

An *action sequence* refers to a specific group of action requests that must occur in a predefined semantic order; for example, raising the foot from the accelerator, moving it across to the brake, and pressing it down on the brake. These actions must occur one after the other and in a specific order. For the purposes of what follows, only the four actions 1…4 in the order 1 → 2 → 3 → 4 comprise a valid *action sequence*.

An *action clique* refers to a group of action requests that may or may not occur in a predefined order but must exclude other specific actions from occurring along with actions within the clique. For example, putting a teabag in a mug, pouring milk into a mug, pouring water into a mug, but *not* putting instant coffee into a mug. The specific order in which tea is made is unimportant, but it is important to not make coffee at the same time. For the purposes of what follows, the four actions 1…4 in any order coupled with the exclusion of action 6 comprise a valid *action clique*.

**Figure 2:**
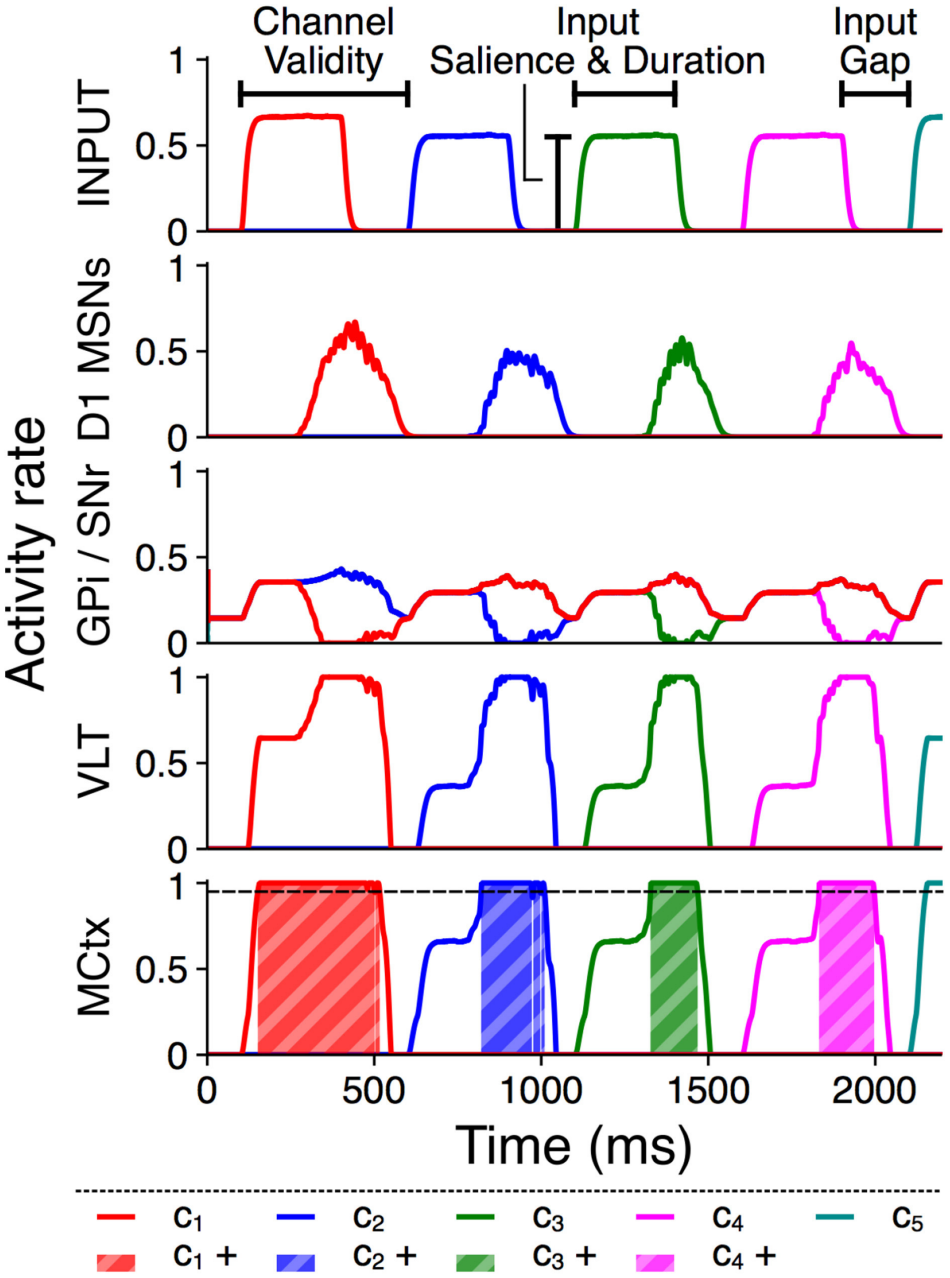
Activity rates of key populations in channels *c*_1_…*c*_5_ in response to a typical action group presentation. Input features and MCtx selection threshold θ = 0.95 are indicated; selection of channel *n* resulting in a positive selection score is highlighted as *c*_*n*_+.

Each action group thus consists of several distinct action requests, and each action request also consists of two phases of activity. The onset of each action request is marked by a transient burst of activity from Poisson generators representing an action channel in sensory cortex, followed by a quiet “gap” period during which the model receives no external stimulus but may sustain selection via feedback within the basal ganglia–thalamocortical loop (Chambers et al., 2005). The transient burst and the subsequent gap together comprise the “valid” period for selection of that action request, which ends at the onset of the next action request. This is comparable to the phasic activity in macaque prefrontal neurons corresponding to saccades during a learned sequence reported by Fujii and Graybiel (2003). Figure 2 illustrates these input features and shows example rate outputs from selected populations.

To ensure consistent initial activation of a series, the first action request in a trial is always active from 100–400 ms at 2,000 spikes/s, and the post-transient gap is 200 ms for all action requests. The input duration and salience of the remaining three action requests in a group varies *between* trials, but each will use the same input salience and duration *within* a trial. Channel *c*_5_ is always active at 2,000 spikes/s following the gap period after the last action request in the group. Every action group is therefore initiated by an input of standard strength, and the remaining action requests within a group all have identical input duration, input salience, and valid length.

In the results that follow we use suprathreshold activity in motor cortex as a quantitative measure of selection. An action channel is determined to be selected when the motor cortex activity rate for that channel is above a threshold of 0.95. A score of 1 is assigned for every simulation timestep of channel *c*_*n*_ validity where that channel is selected, and a score of −1 is assigned for every timestep outside of the valid period where the channel is selected. A score of −1 is also assigned for each timestep any channel is selected at the same time as another, even if this occurs during a valid period. Subthreshold activity in motor cortex at any time scores 0.

The total score is averaged across the duration of the entire action group to give a final overall selection score between −1 and 1. Channel *c*_5_ is not considered part of any action group and so selection of this channel is not scored.

Figure 2 shows a time series of activity rate outputs from key populations in response to presentation of a typical action series with input duration 300 ms and input salience 1,600 spikes/s; the selection score for this presentation is 0.4256. Selection scores from multiple such presentations are aggregated to provide a summary of the overall selection ability of four distinct model configurations: control, diffuse, unidirectional, and pruned.

The *control* configuration does not include any neuropeptides, while in the *diffuse* configuration all GABAergic connections formed by D_1_ MSNs release SP and all GABAergic connections formed by D_2_ MSNs release enkephalin. In the *unidirectional* configuration, SP is released only from connections formed by D_1_ MSNs in channel *c*_*n*_ that target MSNs in channel *c*_*n*+1_ (for *n* < 4). All connections formed by D_2_ MSNs still release enkephalin. The *pruned* configuration is identical to the diffuse configuration except that MSN connections *c*_1_ → *c*_6_ do not release SP. There are no other differences between model configurations, and in all cases the sole effect of neuropeptide release is the facilitation or inhibition of subsequent glutamatergic inputs to the post-synaptic neuron.

## 3. Results

### 3.1. Validation of neuropeptide and hybrid basal ganglia models

#### 3.1.1. Neuropeptides

##### Substance P

Neurophysiological recordings have shown that SP has a presynaptic facilitatory effect on subsequent glutamate inputs to MSNs it targets; in a pair of MSNs A and B where A projects to B, a burst of five spikes over 50 ms in MSN A elicited on average a 14% increase in glutamatergic EPSP amplitude in MSN B 100 ms after the first spike (Blomeley et al., 2009). No facilitation was seen at 50 ms after the first spike, and only residual facilitation was seen after 250 ms.

**Figure 3:**
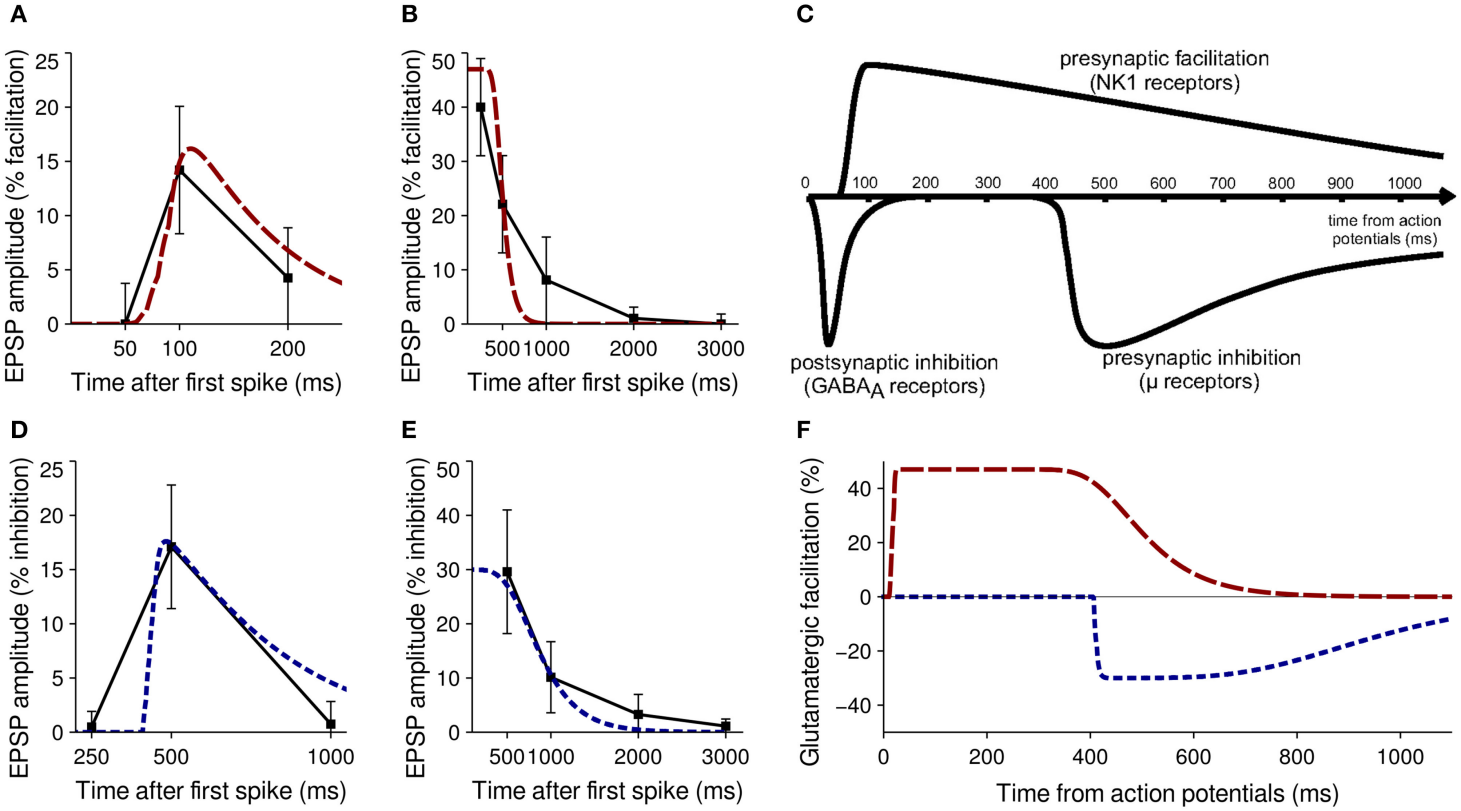
Validation of model neuropeptide performance against neurophysiological data. **(A)** Facilitation of glutamatergic EPSPs by SP after release induced by five spikes from a neighboring MSN *in vitro* (black) and in the model striatum (red). **(B)** Facilitation of glutamatergic EPSPs by SP after release induced by antidromic stimulation of MSNs *in vitro* (black) and in the model striatum (red). **(C)** Qualitative estimate of the timecourse of MSN interactions after a burst of spikes causing a release of GABA, SP (acting on NK_1_ receptors) or enkephalin ((acting on μ- and δ-opioid receptors) from Blomeley and Bracci (2011). **(D)** Inhibition of glutamatergic EPSPs by enkephalin after release induced by five spikes from a neighboring MSN *in vitro* (black) and in the model striatum (blue). **(E)** Inhibition of glutamatergic EPSPs by enkephalin after release induced by antidromic stimulation of MSNs *in vitro* (black) and in the model striatum (blue). **(F)** Timecourse of the effects of SP (red) and enkephalin (blue) on glutamatergic EPSPs after a burst of spikes from model MSNs.

When antidromic spikes were evoked in MSNs, glutamatergic facilitation as a result of SP was ~40% after 250 ms and ~22% after 500 ms (Blomeley et al., 2009). Bath application of SP increased the amplitude of glutamatergic EPSPs by 47% on average (Blomeley and Bracci, 2008). These data formed the primary fitness criteria for the SP model; Figures 3A,B show a comparison of neurophysiological data and model performance for SP, confirming that the phenomenological model captures the facilitatory effect.

##### Enkephalin

Enkephalin has a similar but inhibitory presynaptic effect on glutamatergic inputs to MSNs. In a similar paired–recording experiment, a burst of five spikes in MSN A elicited on average a 17.1% inhibition of glutamatergic EPSP amplitude in MSN B 500 ms after the first spike (Blomeley and Bracci, 2011). No inhibition was seen at 250 ms after the first spike, and minimal inhibition was seen at 1000 ms.

Evocation of antidromic spikes resulted in average inhibition of 29.6% seen after 500 ms, becoming undetectable after 2 s (Blomeley and Bracci, 2011). No data were available on inhibitory effects as the result of bath application. These data formed the primary fitness criteria for the enkephalin model; Figures 3D,E show a comparison of neurophysiological data and model performance for enkephalin, confirming that the phenomenological model captures the inhibitory effect.

##### Model calibration

Calibration of each neuropeptide model thus required the fixing of five variables; the neuropeptide release rise and fall time constants τ_*r*_ and τ_*f*_, the λ and *b*-values for the tuned response curve, and an overall effect multiplier β. The β-value for each neuropeptide was fixed at the maximum observed effect for that neuropeptide and preliminary tuning established reasonable values for τ_*r*_, leaving three free variables for each neuropeptide.

Two calibration experiments were constructed to mimic the setup used in Blomeley et al. (2009) and Blomeley and Bracci (2011); to recreate the paired–recording experiments a single model MSN was provided with five spikes from another model MSN over a 50 ms window, with the resulting effect on subsequent glutamatergic inputs compared to physiological data at 50, 100, and 200 ms after the first spike (for SP) and 250, 500, and 1000 ms after first spike (for enkephalin). To recreate the evocation of antidromic spikes a single model MSN was provided with five spikes from ten separate model MSNs over a 50 ms window. The effect on subsequent glutamatergic inputs was again compared with physiological data to inform model performance. Both calibration experiments were repeated for a wide range of τ_*f*_, λ and *b*-values for each neuropeptide to obtain the best fit to neurophysiological data. Figure 3C shows a qualitative estimate of the timecourse of neuropeptide action from Blomeley and Bracci (2011), and Figure 3F shows the effect of the model neuropeptides after calibration.

#### 3.1.2. Hybrid basal ganglia

**Figure 4:**
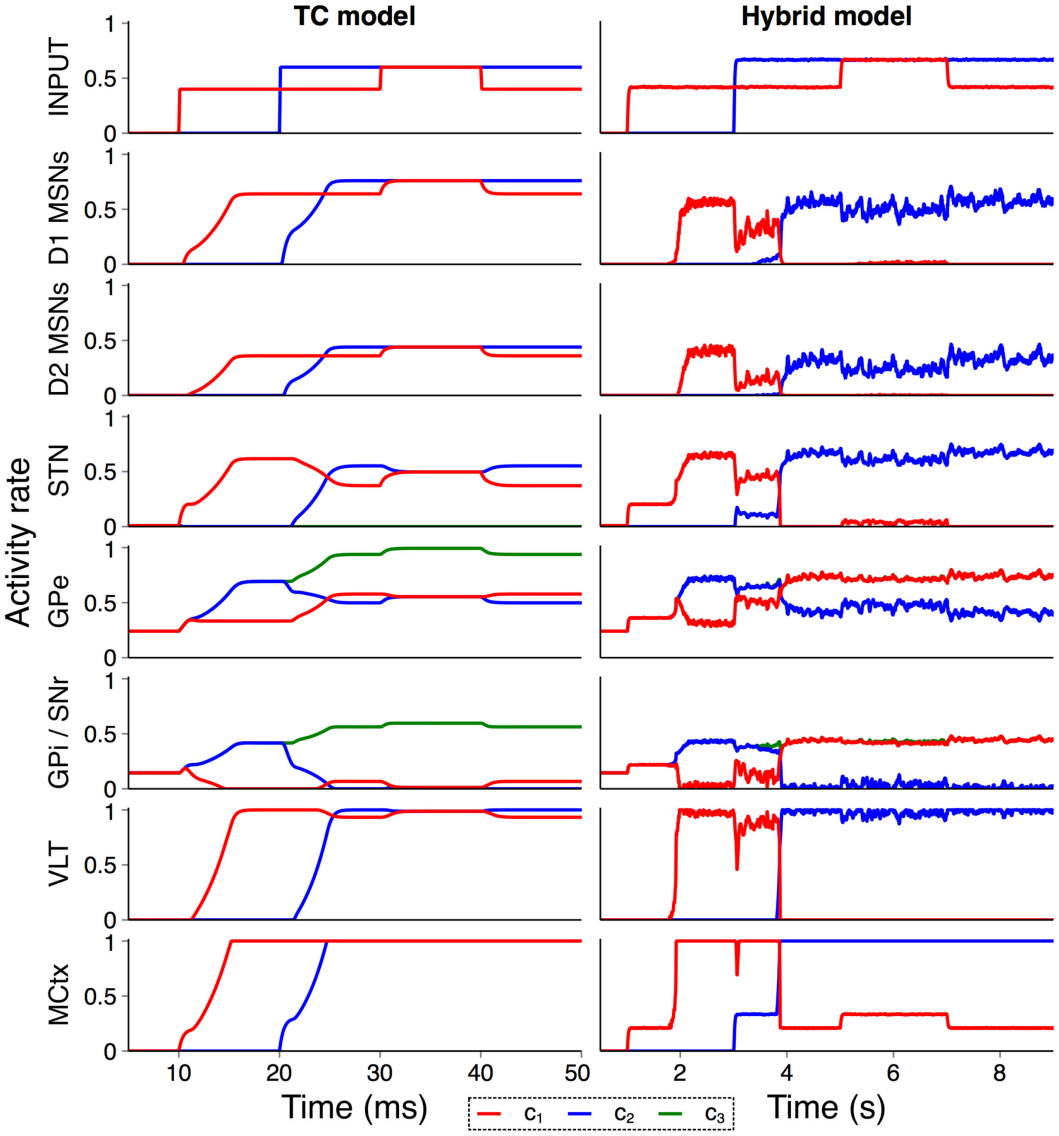
Validation of hybrid model against the TC model. Comparison of activity rates for channels *c*_1_ (red), *c*_2_ (blue), and *c*_3_ (green) in response to a transient input event (Humphries and Gurney, 2002) confirm that population–level behavior of the hybrid model is similar to the rate–coded TC model.

In order to confirm that the hybrid model performs in line with the model from Humphries and Gurney (2002) we recreated an experiment from that paper exploring the model's response to a transient change in input strength. Figure 4 shows the activity rate of all neural populations in the purely rate–coded model compared to the hybrid model. Both models show similar activity rates and overall response to external input, suggesting that the conversion between spiking and rate output is suitably tuned and that the model is behaving in line with expectations. However, structural changes in the hybrid model give rise to some notable differences; MSN spiking dynamics cause a delay between the onset of input activity and striatal output in the hybrid model, and internal connectivity within the striatum that is not present in the TC model prevents simultaneous striatal activity in multiple channels. This also allows the sustained selection of a single action request during the transient event, a feature which required the inclusion of an additional population representing the thalamic reticular nucleus in Humphries and Gurney (2002).

### 3.2. Diffuse neuropeptide connectivity enhances selection of an unordered action series

**Figure 5:**
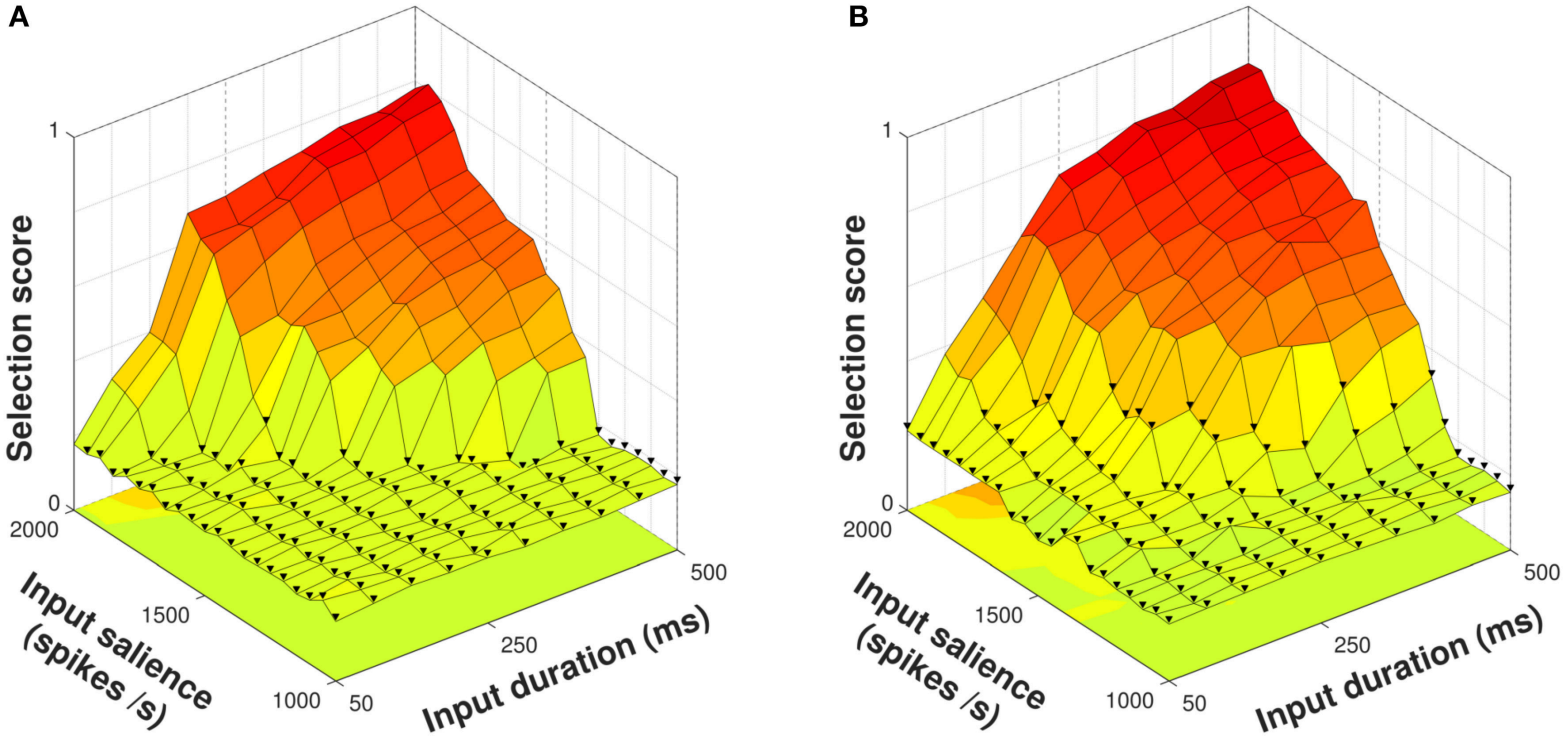
Selection scores for control and diffuse configurations in response to presentation of a four–action series. Trials with fewer than four actions selected indicated with ▾. **(A)** Using the control configuration there is a clear delineation between trials where all action requests are selected and those where some are not. **(B)** The diffuse configuration facilitates selection but does not allow the entire action series to become selected at lower input values.

In order to explore the ability of the model neuropeptides to influence selection of an *action series* — see Section 2.2 — we presented both control and diffuse configurations of the model with a four–action series 1 → 2 → 3 → 4 of varying input duration and salience. Using a control configuration where the model striatum included no neuropeptide projections, the selection score for each trial remained close to zero until input salience rose to at least 1,350 spikes/s for a duration of 500 ms (Figure 5A). This rise in selection score corresponded to successful selection of the entire series, which occurred at lower durations as the input salience increased and shows that both input features have an impact on selection of an action request and implying that they are to some degree interchangeable. The mean selection score for all action series presentations using the control configuration was 0.3273.

Using the diffuse configuration resulted in an increase of the mean selection score to 0.3788 (Figure 5B); however, this did not correspond to the series as a whole being reliably selected at lower input salience or duration values. Instead, action requests that were successfully selected using the control configuration were selected for a longer duration, and some action requests that were just below the threshold for selection in the control configuration were able to become selected.

**Figure 6:**
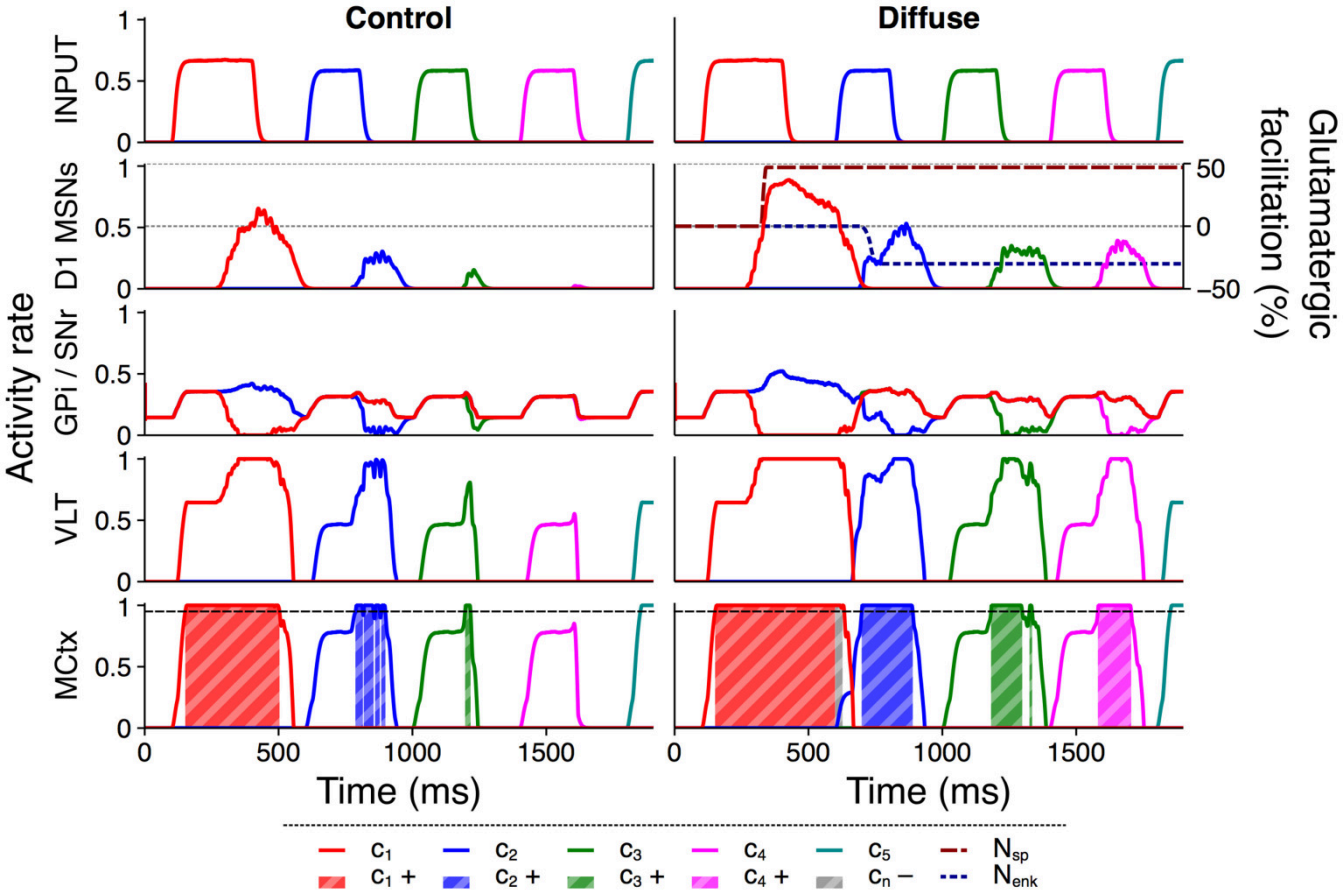
Diffuse neuropeptide connectivity facilitates selection of above– or near–threshold action requests. Activity rates of key populations in channels *c*_1_…*c*_5_ following presentation of an action series with input duration 200 ms and input salience 1,700 spikes/s. MCtx selection threshold θ = 0.95 is indicated; selection of channel *n* resulting in a positive selection score is highlighted as *c*_*n*_+. Selection of any channel resulting in a negative score is highlighted as *c*_*n*_−. D_1_ MSN activity is overlaid with neuropeptide glutamatergic effects *N*_sp_ and *N*_enk_.

Figure 6 illustrates this facilitation with a comparison of selected rate outputs following presentation of a single action series with input duration 200 ms and salience 1,700 spikes/s to both configurations. Under the control configuration the selection of channel *c*_2_ was intermittent, channel *c*_3_ was selected very briefly, and channel *c*_4_ was entirely unselected. The selection score for this trial was 0.2491. Using the diffuse configuration, the selection of channels *c*_2_ and *c*_3_ was stronger, and activity in channel *c*_4_ was raised above the threshold for selection. The extended selection of channel *c*_1_ also resulted in a brief period in which selection was sustained into the onset of channel *c*_2_, and the onset of enkephalin–based inhibition caused a brief dip in striatal activity during the transient burst of activity to channel *c*_2_. As a result of the improved selection under this configuration the score for this trial was 0.4953 and the entire series was successfully selected.

Because all D_1_ MSNs release SP irrespective of their targets in the diffuse configuration, facilitation of an action request using this configuration arises from both within– and between–channel effects. Once an action request is selected, SP released from that action channel feeds back into the same channel and encourages the sustained selection of that channel, as evidenced by the longer periods of selection for channels *c*_1_…*c*_3_. In addition, selection of any request releases SP that projects to every other channel and facilitates otherwise subthreshold action requests, shown by the successful selection of channel *c*_4_ and the more rapid selection of channel *c*_2_.

Thus, diffuse neuropeptide connectivity improved the quality of selection for action requests already above threshold and in some cases was able to raise previously subthreshold action requests to a suprathreshold level. With only a few exceptions, however, this did not allow for entire action series to be selected given inputs of lower strength.

### 3.3. Unidirectional substance P and diffuse enkephalin enhance sequence–specific selection

The distributed SP projections in the diffuse configuration necessarily makes it unable to preferentially facilitate one action request over another, and therefore potentially unable to distinguish between ordered and disordered presentations of a semantically ordered *action sequence*. To investigate the performance of a unidirectional SP configuration in this regard we presented an action sequence in both ordered (1 → 2 → 3 → 4) and disordered (4 → 3 → 2 → 1) states to models using control, diffuse and unidirectional configurations. Because an action sequence is semantically ordered, a higher selection score corresponds to greater selection of the ordered presentation and greater inhibition of the disordered presentation.

**Figure 7:**
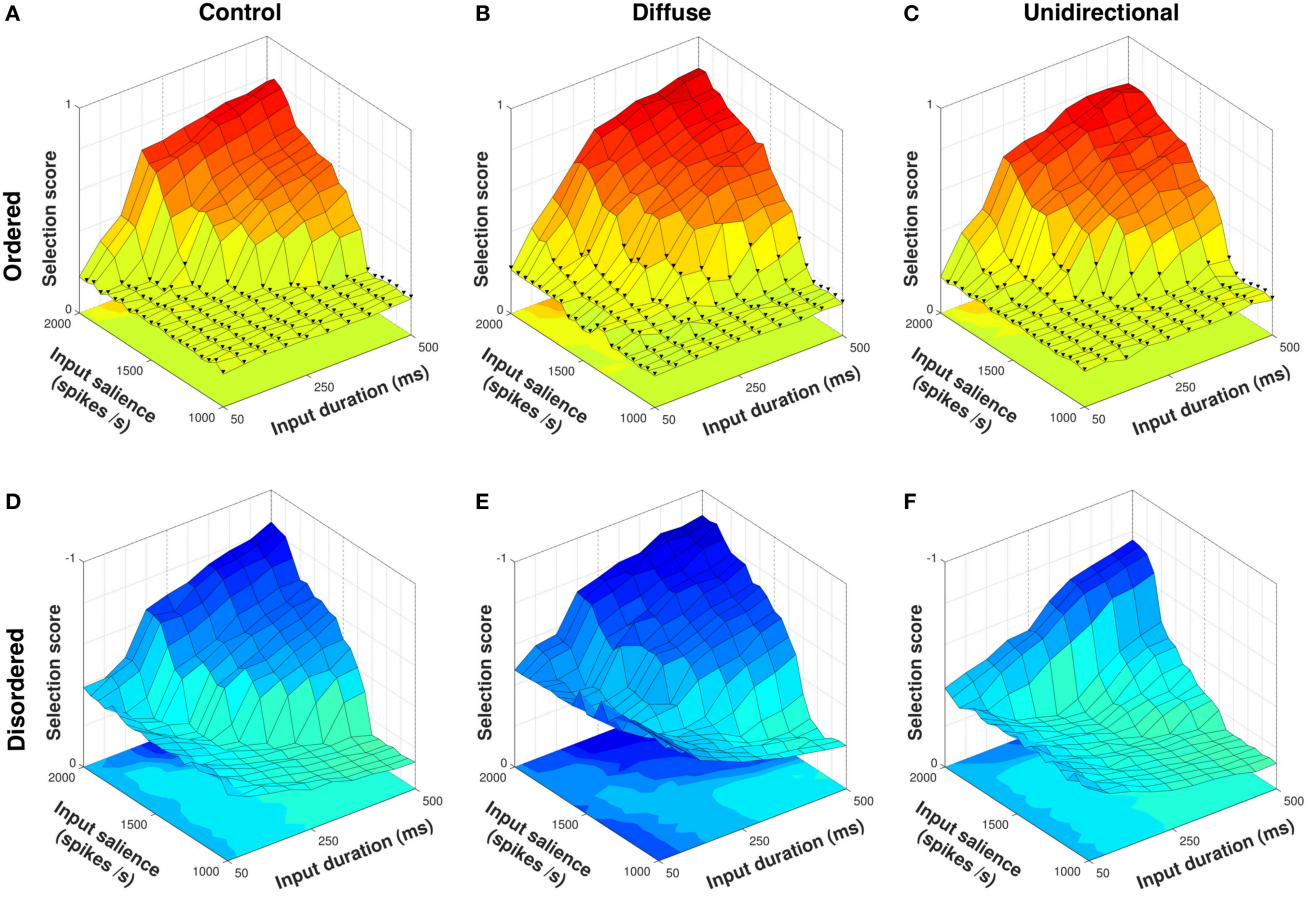
Selection scores in response to ordered and disordered presentations of a four–action sequence. Ordered presentations with fewer than four actions selected indicated with ▾; disordered presentations shown with Z–axis inverted. Presentation of an ordered sequence to the diffuse configuration **(B)** results in facilitation of selection compared to control **(A)**. However, because these configurations are sequence–agnostic, disordered presentations to control **(D)** and diffuse **(E)** configurations result in erroneous selection across a wide range of input values. Compared to control, the unidirectional configuration both facilitates selection of the ordered presentation **(C)** and inhibits selection of the disordered presentation **(F)**.

Figure 7 shows selection scores for ordered and disordered sequence presentations to control, diffuse, and unidirectional configurations. Because the first two configurations are necessarily sequence–agnostic their response to both presentations is identical to an arbitrarily–ordered series as in Figure 5; the difference in selection scores between ordered and disordered presentations is thus due to scoring the disordered presentation according to the correct semantic order rather than a change in model performance.

The unidirectional configuration conferred a slight advantage to selection of an ordered sequence presentation over control, with a mean selection score of 0.3542 (Figure 7C) compared to 0.3273 for control (Figure 7A). This was lower than the mean selection score of 0.3788 for the diffuse configuration (Figure 7B), implying that the difference in neuropeptide projection architecture in these configurations impacted the facilitation of selection. In the unidirectional configuration, only SP projections of the form *c*_*n*_ → *c*_*n*+1_ are permitted, with the result that facilitation of an action request can only occur if the previous request in the sequence is selected. Thus, within–channel feedback facilitation cannot occur in the unidirectional configuration, resulting in a lower mean selection score than the diffuse configuration in response to an ordered sequence presentation.

However, the distributed between–channel facilitation present in the diffuse configuration caused erroneous selection in response to a disordered presentation (Figure 7E). The inability to inhibit (or prevent facilitation of) undesired action requests resulted in a mean selection score of −0.5005 for the diffuse configuration compared to −0.3654 for control (Figure 7D). Conversely, the unidirectional configuration was able to actively inhibit the disordered requests, resulting in a higher mean selection score of −0.2913 (Figure 7F).

**Figure 8:**
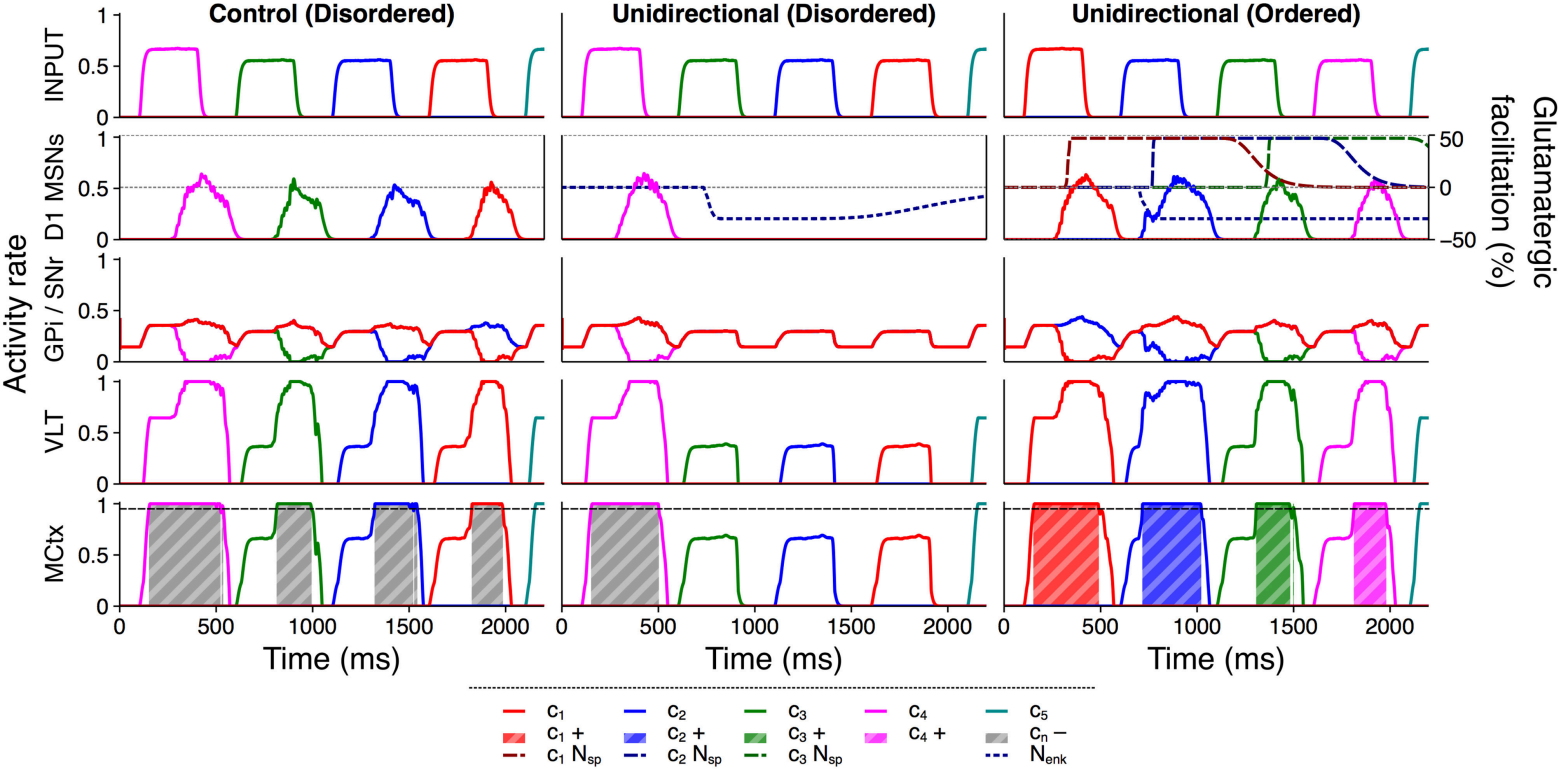
Active inhibition of disordered action requests requires enkephalin and is counteracted by substance P. Activity rates of key populations in channels *c*_1_…*c*_5_ following presentation of an ordered or disordered action sequence with input duration 300 ms and input salience 1,600 spikes/s. Interpretation of graphical elements as in Figure 6, with the addition of channel–specific SP effects *c*_*n*_*N*_sp_.

Figure 8 illustrates how between–channel SP and diffuse enkephalin interacted in the unidirectional configuration to facilitate selection and inhibition of ordered and disordered action requests respectively. In the control configuration, each individual action request was salient enough to become (erroneously) selected without additional facilitation. However, in the disordered presentation to the unidirectional configuration the selection of the first action request released sufficient enkephalin to inhibit selection of the remaining requests; enkephalin's long timecourse enabled the inhibition of multiple successive action requests in the absence of SP–based facilitation. The ordered presentation to the unidirectional configuration shows that the presence of between–channel SP was sufficient to counteract enkephalin–based inhibition and enabled ordered action requests to be selected normally or even more strongly than in the control configuration.

The presence of distributed enkephalin projections therefore made the selection of subsequent action requests contingent on the presence of SP; inhibition of other requests became a default counteracted by SP influx. Not only was selection of an ordered sequence improved in response to the unidirectional facilitation of action requests, but the semantic ordering of the sequence was protected by the inhibition of disordered action requests.

### 3.4. Selectively pruned substance P enhances separation of action cliques

We have shown that the diffuse configuration confers the strongest advantage to selection of unordered action requests by utilizing both within– and between–channel SP projections to facilitate action requests, and that the unidirectional configuration allows active inhibition of disordered requests by limiting facilitation to ordered between–channel projections. We sought to explore if selective inhibition of undesired requests could also allow for useful structuring of action groups. We therefore presented all four model configurations with action requests in the order 1 → 6 → 2 → 3 → 4, where action channels *c*_1_…*c*_4_ formed an *action clique* and channel *c*_6_ represented an undesired distractor action.

**Figure 9:**
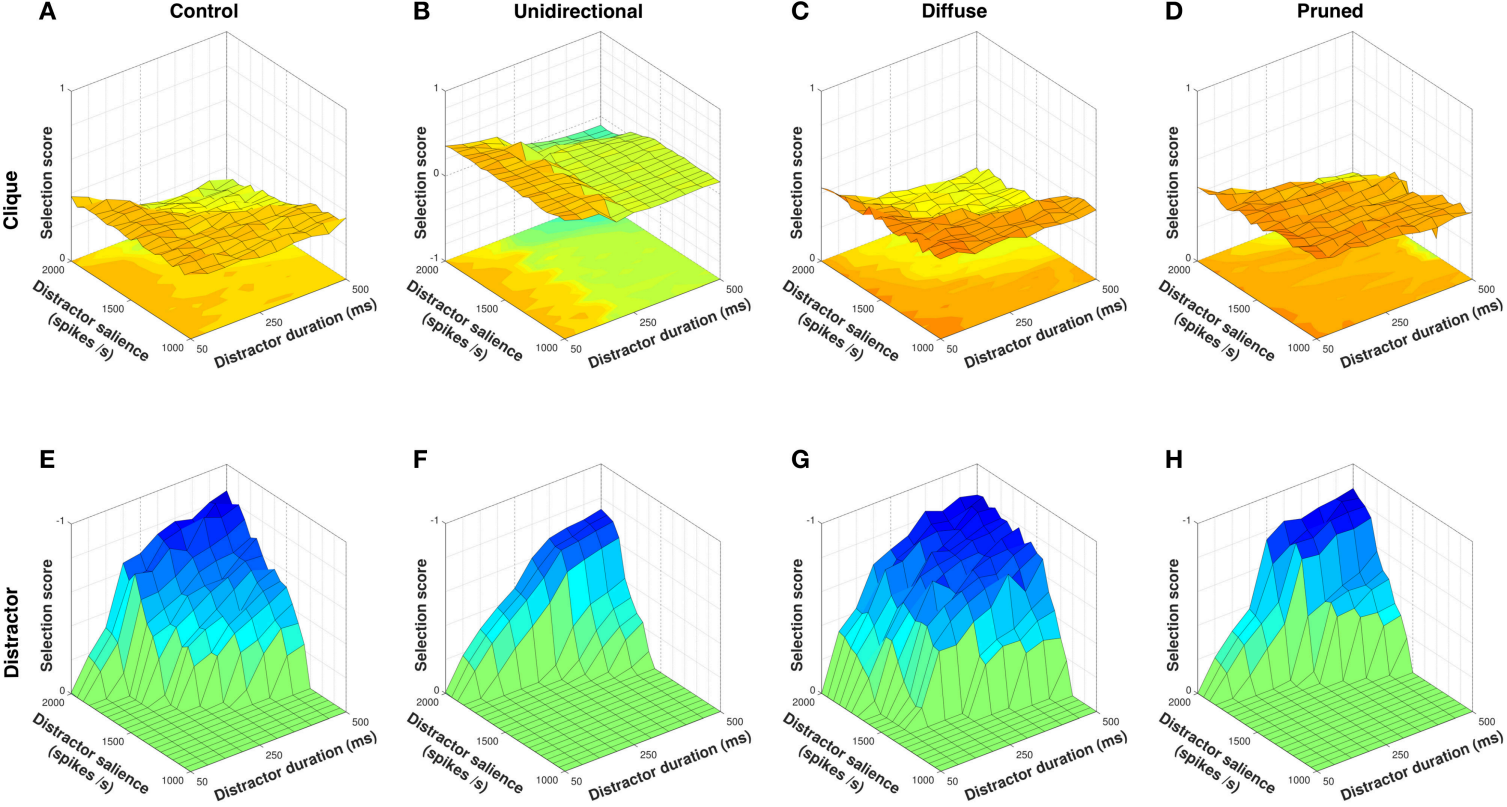
Selection scores for clique and non-clique actions for all model configurations. Non-clique scores shown with Z–axis inverted. The control configuration is moderately able to select clique action requests **(A)** but is unable to inhibit selection of the non-clique distractor **(E)**. The unidirectional configuration is able to actively inhibit the distractor **(F)** but is less able to select clique action requests when distractor duration is high **(B)**. The diffuse configuration facilitates selection of clique action requests **(C)** but also facilitates selection of the distractor **(G)**. The pruned configuration is able to selectively inhibit the distractor **(H)** and facilitate selection of the clique across a wider range of distractor input values **(D)**.

In exploring the model's response to an action clique, the input duration and salience of each action request in the clique was fixed at 300 ms and 1,600 spikes/s respectively, and only the salience and duration of the non-clique distractor was varied. Figure 9 shows clique and non-clique selection scores for each model configuration.

In line with results from the disordered sequence presentation (Figure 7F), the unidirectional configuration was able to inhibit the distractor request (Figure 9F) better than the control condition (Figures 9E) due to the presence of diffuse enkephalin that was not counteracted by a directed SP projection. Mean distractor selection score for the unidirectional configuration was −0.0986, compared to −0.2090 for control. However, the ability of the unidirectional configuration to facilitate clique action requests dropped off to levels below that of control as the duration of the distractor rose above ~250 ms (Figures 9A,B). Mean clique selection score for the unidirectional configuration was 0.1919, compared to 0.2943 for control. Closer examination of the neuropeptide dynamics revealed that the longer timecourse of enkephalin coupled with the lack of within–channel feedback facilitation resulted in the facilitatory effect of between–channel SP falling off before the onset of channel *c*_2_, resulting in effective inhibition of the remaining clique action requests.

Also in line with results from the action series presentation (Figure 5B), the diffuse configuration was able to select clique action requests better than control (Figure 9C) due to the combination of within– and between–channel facilitation, resulting in a mean selection score of 0.3415. However, the diffuse SP projections caused equivalent facilitation of the distractor request (Figure 9G) resulting in a mean distractor selection score of −0.3112 and thus no effective separation of clique from non-clique requests.

The pruned configuration was the only neuropeptide projection scheme that performed better than control at both selection of the clique (Figure 9D) and inhibition of the distractor (Figure 9H). Mean clique selection score for the pruned configuration was 0.3718, and mean distractor selection score was −0.1314. The removal of SP projections from channel *c*_1_ → *c*_6_ inhibited the non-clique action request and allowed within–channel SP feedback in channel *c*_1_ to sustain selection of that channel until the delayed onset of channel *c*_2_, resulting in strong selection of the clique even with a high duration distractor. The inclusion of feedback SP projections within channel *c*_6_ also allowed for strong selection of the distractor request when it was salient enough to overcome the diffuse inhibition, and therefore resulted in a greater contrast between inhibition and selection of non-clique action requests.

Selective removal of channel–specific SP projections therefore allowed for inhibition of individual action requests that enhanced both the separation of distinct action cliques and facilitation of selection within a given clique. Furthermore, because this was dependent on the removal of specific SP projections, the resulting inhibition is context–sensitive and potentially allows for the suppression of distinct distractors in different situations with only minimal changes to the SP network.

## 4. Discussion

We have created novel phenomenological models of the effects of two striatal neuropeptides based on neurophysiological data (Blomeley et al., 2009; Blomeley and Bracci, 2011) and integrated these into a hybrid model of the basal ganglia based on previous work (Humphries and Gurney, 2002; Humphries et al., 2010). By exploring several neuropeptide connectivity configurations and presenting the model with groups of action requests we have shown that inclusion of these neuropeptides improves the model's ability to both select and reject action requests, and that pruning the SP network improves the model's ability to selectively inhibit actions that are disordered or undesired. In addition, we have shown that the interaction of SP and enkephalin is of key importance to the effective inhibition of undesired action requests.

### 4.1. Action and interaction of neuropeptides

Presenting the model with multiple groups of action requests allowed us to examine the effects of SP and enkephalin within and between MSN populations representing each channel. Presentation of an action series with no preferred semantic order revealed that SP facilitates action requests in two distinct ways. Firstly, SP acts within an action channel to promote its continued selection by facilitating glutamatergic inputs from MCtx regions representing the currently active channel. This leads to sustained inhibition of GPi/SNr activity and thereby permits continued activity in the region of the MCtx–VLT loop representing the current channel, closing the loop. Secondly, SP acts between action channels by facilitating glutamatergic inputs from cortical regions representing other action requests, raising their effective salience and potentially enabling the selection of otherwise subthreshold requests (Figure 6). The combination of these two effects allows the diffuse release of striatal neuropeptides to facilitate selection of action requests within an unordered series.

The presentation of a disordered action sequence showed that this generalized facilitation can be a detriment to the inhibition of undesired action requests (Figure 7E). When SP release is restricted to MSN connections targeting MSNs representing the next sequential action, enkephalin can actively inhibit disordered requests and prevent the erroneous selection of a disordered sequence (Figure 7F). This enhanced inhibitory ability is not present in the absence of enkephalin (Figure 7D).

We also showed that the between–channel effects of SP are sufficient to counteract this inhibition and allow the selection of a correctly–ordered sequence with greater strength than if no neuropeptides were present (Figure 8). Therefore, a transition between two actions *A* and *B* within the active timecourse of enkephalin is contingent on either the presence of an SP projection *A* → *B* or an extremely salient request for action *B*.

Presentation of an action clique with an additional distractor request showed that by selectively pruning SP connections, the ability of the model to both select an action clique and inhibit distractions is improved with minimal changes to the SP network (Figure 9). Although diffusely–connected SP and enkephalin facilitate action requests and raise their effective salience above control levels (Figure 6), the more compelling result may be the inhibition of otherwise salient action requests that occurs when SP projections between MSN populations are removed (Figure 8).

Thus, the overall picture is of diffuse enkephalin release causing broad inhibition of incoming action requests, counteracted by targeted SP release that facilitates the initial and sustained selection of specific requests. This agrees with results from serial selection tasks in rats showing that increased activation of D_1_ receptors reduced selection accuracy, while increased activation of D_2_ receptors increased perseverative responses (Domenger and Schwarting, 2006; Agnoli et al., 2013). Adjusting the strength of SP projections between MSN populations may give rise to striatal connectivity supporting ordered action sequences or distinct action cliques that also enhances the contrast between selected and inhibited requests, similar to the “unsharp mask” model of striatal processing proposed by Stocco and Lebiere (2013) arising from the enhancement of lateral inhibition combined with localized facilitation.

### 4.2. Substance P and learning

The ability of the unidirectional and pruned configurations to selectively inhibit an otherwise salient action request in favor of an alternative suggests that learning of action sequences or cliques may involve plasticity of striatal SP connections. Plasticity need not directly promote the selection of related action requests but could raise their relative salience by reducing the level of SP connections targeting MSNs representing potential distractions, thereby inhibiting undesired requests.

We are as yet unaware of any direct evidence for SP plasticity within the striatum, though SP has been previously implicated in the facilitation of learning (Huston and Hasenöhrl, 1995; Hasenöhrl et al., 2000) and in affecting motivational aspects of reward (Murtra et al., 2000). Skill learning has also been shown to result in a relative increase in D_2_ MSN activity (Yin et al., 2009) that could plausibly correlate with refinement of SP connectivity. Furthermore, a pattern of preferentially within–channel and within–sequence SP projections combined with diffuse enkephalin projections is consistent with closed– and open–loop reverberations resulting from stimulation of D_1_ or D_2_–type receptors respectively as reported by Carrillo-Reid et al. (2011).

We have explored only a basic example of selective inhibition; as it is context–sensitive and channel–specific, more complex modifications could allow for groups of MSNs to represent multiple overlapping cliques simultaneously. Patterned SP connectivity may allow the striatum to incorporate probabilistic links between component actions that could represent a neurological basis for “chunking” (Graybiel, 1998) and higher–order hierarchical groups (Balleine et al., 2015).

MSN populations thus delineated by patterned SP connections may act as “local controllers” (Graybiel and Grafton, 2015) of sequence chunks that facilitate the efficient selection of ordered cortical inputs; indeed, striatal activity during motor chunking has been shown to correspond to the concatenation of sequence elements while cortical activity correlates with their segmentation (Wymbs et al., 2012). It would therefore not be correct to say that sequences are “stored” within the striatum. While the striatum is heavily involved in the chunking of cortical inputs (Graybiel, 2008; Jin et al., 2014), it also relies on cortical inputs to provide additional information about the order, timing, duration and salience of individual actions. Sequence execution is therefore a result of the coordinated interaction of both structures.

We may postulate a role for SP as a modulator of dopamine in the formation of sequence chunks. It has recently been reported that SP may modulate dopamine transmission differently according to neuronal location within the striosomal–matrix axis (Brimblecombe and Cragg, 2015) and movement chunking itself has been shown to be dependent on dopamine (Tremblay et al., 2010). The question of whether SP projections in striatum are plastic as part of sequence learning — and if so, how this plasticity is mediated — should prove enlightening.

### 4.3. Clinical implications

The impact of striatal neuropeptides on action selection has some potential clinical implications. Huntington's disease causes the degeneration of both D_1_ and D_2_ MSNs (Turjanski et al., 1995; Weeks et al., 1996) but preferentially impacts D_2_–expressing neurons (Richfield et al., 1995; Mitchell et al., 1999). It has been shown that Huntington's disease specifically impairs the learning of motor sequences (Willingham and Koroshetz, 1993); one plausible explanation is that a deficit in enkephalin release resulting from the degeneration of D_2_ MSNs leads to an inability to selectively facilitate sequential action requests and thus to integrate semantic relationships into the structure of striatum.

Huntington's disease also has well–established impacts on cognitive function that often manifest long before the first indications of motor problems (Paulsen et al., 2001; Duff et al., 2010). Because the basal ganglia also process signals from limbic and associative functional territories, we may plausibly speculate that striatal neuropeptides play a similar role in action selection across these cognitive domains. Enkephalinergic degeneration causing an inability to inhibit undesired action requests could therefore also potentially explain the increase in impulsivity and risk–taking behaviors seen in Huntington's disease (Kalkhoven et al., 2014; El Massioui et al., 2016).

Conversely, the increased apathy seen in Huntington's disease seems contrary to expectations from a loss of inhibitory signaling. Heightened apathy is correlated to decline in both cognitive and motor function (Thompson et al., 2002; Baudic et al., 2006; Naarding et al., 2009), and it has been suggested that this apathy is a manifestation of an overall reduction in “drive and motivation” (Hamilton et al., 2003) resulting from these problems. Some preliminary success in slowing cognitive decline in Huntington's disease has been seen following cholinergic interventions (de Tommaso et al., 2004; Morton et al., 2005), suggesting that the role of striatal interneurons and neurotransmitters absent from the current model may be important for a fuller understanding of this condition.

The ADHD–like symptoms of mice lacking functional NK_1_ receptors (Yan et al., 2009; Porter et al., 2015) may also be partially explained by a lack of within–channel feedback facilitation to sustain selection of a given action. An inability to sustain selection without external stimuli could cause changes in corticostriatal activity that may reflect “an increased need for or reliance on vigilance or sustained visual attention” reported in children with ADHD (Durston et al., 2003). Definitive conclusions about the mechanisms underlying specific pathologies is beyond the scope of this study, however.

### 4.4. Concluding remarks

Several issues remain unaddressed. To avoid “edge effects” and maintain a consistent density of connections the striatal model used here ignores topological relationships between neurons and resembles the *random* model from Tomkins et al. (2014). Any potential effects resulting from the physical organization of MSNs representing specific action channels, or from second–order organizational features are therefore beyond the capabilities of the current model. We also do not attempt to model the complex connectivity and interactions of the patch and matrix components of the striatum. Furthermore, as the rate–coded basal ganglia–thalamocortical loop model is taken directly from Humphries and Gurney (2002) it necessarily inherits the lack of thalamic burst firing and interneurons from that model.

However, it is apparent that the interaction of neuropeptides allows for an additional layer of computational complexity within the striatum. The facilitatory effects of SP imply an unexplored co-operative role for MSNs that warrants further investigation, especially in regards to the development of patterned SP connectivity. Exploration of the computational role of striatal interneurons not modeled here should also prove illuminating; SP has been found to modulate the activity of cholinergic interneurons (Govindaiah et al., 2010), and the responses of tonically active interneurons are strongly correlated with the likelihood of a behavioral response to a stimulus (Blazquez et al., 2002). Future additions to models of neuropeptide interactions will doubtless reveal new striatal functions.

## Author contributions

Each author contributed to the submission of this report. DB performed neural modeling, results analysis, and write-up of drafts. KG provided assistance and guidance with modeling and results analysis and gave suggestions on report structure as well as editing suggestions and portions of the text. PO and EB provided feedback, suggestions and guidance during modeling as well as suggestions regarding text content and input regarding editing. All authors have approved the final version of the text.

### Conflict of interest statement

The authors declare that the research was conducted in the absence of any commercial or financial relationships that could be construed as a potential conflict of interest.
