# Supplementary material for: Striatal Neuropeptides Enhance Selection and Rejection of Sequential Actions
Source: Front Comput Neurosci. 2017 Jul 27;11:62. doi: 10.3389/fncom.2017.00062 (PMC5529366; doi:10.3389/fncom.2017.00062)
Supplement: Supplementary file 1 [file Table1.PDF]

Supplementary Material: Striatal neuropeptides enhance selection and rejection of sequential actions

David Buxton*, Enrico Bracci, Paul G. Overton and Kevin Gurney
*Correspondence:
David Buxton:
d.r.buxton@sheffield.ac.uk

The following is a complete technical description of both the spiking striatal microcircuit model (including phenomenological neuropeptide models) and the basal ganglia–thalamocortical loop model, as per the format suggested by Nordlie et al. (2009).

Table S1. Striatal microcircuit: Summary

Striatal microcircuit: Model summary	
**Populations**	Three: D1 MSNs, D2 MSNs, and FSIs
**Topology**	—
**Connectivity**	GABAergic: All-to-all, pruned according to probability profile<br>Neuropeptide: GABAergic, pruned according to probability profile<br>To basal ganglia–thalamocortical loop: Fixed channel–convergent
**Neuron model**	Modified Izhikevich point neurons with reset condition and fixed voltage threshold
**Channel models**	—
**Synapse model**	Conductance-based single–exponential with dopamine and neuropeptide modifiers
**Plasticity**	—
**Input**	Independent Poisson spike sources representing sensory cortex project to each neuron<br>MCtx output from BG–thalamocortical loop
**Measurements**	Raster log of spikes and converted rate output

Table S2. Striatal microcircuit: Populations

Striatal microcircuit: Populations			
**Name**	**Type**	**Size**	**Organisation**
D1 MSNs (d1)	Modified Izhikevich	3000	Six action channels $c_1 \ldots c_6$ of 500 sequential neurons
D2 MSNs (d2)		3000	
FSIs (fs)	Modified nonlinear Izhikevich	60	—

Table S3. Striatal microcircuit: Connectivity

**Striatal microcircuit: Connectivity**	
**Connection**	**Algorithm**
MSN $\to$ MSN (GABA)	For d1 $\to$ d1, d1 $\to$ d2, d2 $\to$ d1, d2 $\to$ d2:<br>1. Create all–to–all connections<br>2. Assign each connection value $R \sim U([0,1])$<br>3. Connection probability $P = \frac{\text{Number of afferent connections}^*}{\text{Number of target neurons}}$<br>4. Remove connections where $R \leq P$
MSN $\to$ MSN (SP)	For d1 $\to$ d1, d1 $\to$ d2:<br>Diffuse: All GABA connections co-release SP<br>Pruned: All GABA connections except $\mathrm{d1}_1 \to \mathrm{d1}_6$, $\mathrm{d1}_1 \to \mathrm{d2}_6$ co-release SP<br>Unidirectional: Only GABA connections $\mathrm{d1}_c \to \mathrm{d1}_{c+1}$, $\mathrm{d1}_c \to \mathrm{d2}_{c+1}$ where $c < 4$ co-release SP
MSN $\to$ MSN (ENK)	For d2 $\to$ d1, d2 $\to$ d2:<br>All GABA connections co-release enkephalin
FSI $\to$ MSN	For fs $\to$ d1, fs $\to$ d2:<br>As MSN $\to$ MSN (GABA)
FSI $\to$ FSI (GABA)	For fs $\to$ fs:<br>As MSN $\to$ MSN (GABA)
FSI $\to$ FSI (GAP)	For fs $\to$ fs:<br>As MSN $\to$ MSN (GABA) except:<br>1. Begin with all fs $\to$ fs synapse connections
*Values for expected number of afferent connections taken from Table 5, Humphries et al. (2010)	

Table S4. Striatal microcircuit: Neuron models

**Striatal microcircuit: Neuron models**		
**Name**	**Dynamics**	**Modifications**
D1 MSN	$C\dot{v} = k(v - v_r)(v - v_t) - u + I$<br>$\dot{u} = a\left[b(v - v_r) - u\right]$<br>$I = I_{\text{ampa}} + I_{\text{gaba}} + B(v)I_{\text{nmda}}$	$v_r \leftarrow v_r(1 + K\phi_1)$<br>$d \leftarrow d(1 - L\phi_1)$
D2 MSN	Reset: if $v > v_{\text{peak}}$ then $v \leftarrow c, u \leftarrow u + d$	$k \leftarrow k(1 - \alpha\phi_2)$
FSI	$C\dot{v} = k(v - v_r)(v - v_t) - u + I$<br>$\dot{u} = \begin{cases} -au & \text{if } v < v_b \\ -a\left[b(v - v_b)^3 - u\right] & \text{if } v \geq v_b \end{cases}$<br>$I = I_{\text{ampa}} + I_{\text{gaba}}$<br>Reset: if $v > v_{\text{peak}}$ then $v \leftarrow c, u \leftarrow u + d$	$v_r \leftarrow v_r(1 - \eta\phi 1)$
Rate–to–spike	Each timestep, emit spike if $y_i^{\text{mc}} r_{\max} \tau_{\text{bg}} > P$	$P \sim U([0,1])$

Table S5. Striatal microcircuit: Synaptic models

**Striatal microcircuit: Synaptic models**	
**Type**	**Dynamics**
Synapse	$I_z = \bar{g}_z h_z (E - v)$<br>$\dot{h}_z = \frac{-h_z}{\tau_z}$, and $\quad h_z(t) \leftarrow h_z(t) + \left[1 - \frac{h_z(t)}{N_z}\right] S_z(t)$<br>Where z is AMPA, GABA or NMDA and $S_z(t)$ is the number of presynaptic spikes arriving at receptors for z at time t<br>$B(v) = \frac{1}{1 + \frac{[\mathrm{Mg}^{2+}]_0}{3.57} \exp(-0.062v)}$
Neuropeptide	$A_p(t) = \sum_i S_p \left[\exp\left(\frac{-(t - t_i)}{\tau_f^p}\right) - \exp\left(\frac{-(t - t_i)}{\tau_r^p}\right)\right]$<br>$N_p(t) = \beta_p \left[1 - \exp\left(-\frac{A_p(t)}{\lambda_p}\right)^{b_p}\right]$<br>Where p is SP or enkephalin and S_p is the number of spikes causing a release of neuropeptide p
Gap junction	$\tau \dot{v}_{ij}^* = (v_i - v_{ij}^*) + (v_j - v_{ij}^*)$
**Name**	**Modifications**
D1 MSN	$I_{\mathrm{nmda}} = I_{\mathrm{nmda}}(1 + \beta_1 \phi_1)$
D2 MSN	$I_{\mathrm{ampa}} = I_{\mathrm{ampa}}(1 - \beta_2 \phi_2)$
D1 & D2 MSN	$I_z = I_z \left[1 + N_{\mathrm{sp}}(t - \tau_d^{\mathrm{sp}})\right] \left[1 - N_{\mathrm{enk}}(t - \tau_d^{\mathrm{enk}})\right]$<br>Where z is AMPA or NMDA
FSI	$I_{\mathrm{gaba}} = I_{\mathrm{gaba}}(1 - \epsilon_2 \phi_2)$

Table S6. Striatal microcircuit: Inputs

**Striatal microcircuit: Inputs**			
**Input**	**Targets**		
	D1 MSN	D2 MSN	FSI
Sensory cortex (sc)	$\mathrm{sc}_c^i \rightarrow \mathrm{d1}_c^i, 1 \leq i \leq 500$	$\mathrm{sc}_c^i \rightarrow \mathrm{d2}_c^i, 1 \leq i \leq 500$	$\mathrm{sc}_c^i \rightarrow \mathrm{fs}^i, 1 \leq i \leq 60$
MCtx (mc)	$\mathrm{mc}_c \rightarrow \mathrm{d1}_c^i, 1 \leq i \leq 500$	$\mathrm{mc}_c \rightarrow \mathrm{d2}_c^i, 1 \leq i \leq 500$	$\mathrm{mc}_c \rightarrow \mathrm{fs}^i, 1 \leq i \leq 60$

Table S7. Striatal microcircuit: MSN properties

**Striatal microcircuit: MSN properties**		
**Param.**	**Value**	**Source**
a	0.01	Mahon et al. (2000)
b	-20	Izhikevich (2007)
c	-55mV	
k	1	
v_r	-80mV	
v_{peak}	40mV	
C	15.2pF	Humphries et al. (2009)
d	91	
K	0.0289	
L	0.331	
v_t	-29.7mV	
α	0.032	

Table S8. Striatal microcircuit: FSI properties

**Striatal microcircuit: FSI properties**		
**Param.**	**Value**	**Source**
a	0.2	Izhikevich (2007)
b	0.025	
d	0	
k	1	
v_{peak}	25mV	
v_b	-55mV	
C	80pF	Tateno et al. (2004)
c	-60mV	
v_r	-70mV	
v_t	-50mV	
ϵ	0.625	Fitted to Gorelova et al. (2002)
η	0.1	Fitted to Bracci et al. (2002)

Table S9. Striatal microcircuit: Synapse properties

Striatal microcircuit: Synapse properties		
**Parameter**	**Value**	**Source**
$E_{\text{ampa}}, E_{\text{nmda}}$	0mV	Moyer et al. (2007)
E_{gaba}	-60mV	
τ_{ampa}	6ms	
τ_{nmda}	160ms	
τ_{gaba}	4ms	
τ_{bg}	0.1ms	Tuning (see text)
$\tau_{\text{fs-gap}}$	5ms	Fitted to Galarreta and Hestrin (1999)
$[MG^{2+}]_0$	1mM	Jahr and Stevens (1990)
g_{ampa} Ctx–MSN	0.4nS	Tomkins et al. (2014)
g_{ampa} Ctx–FSI	1nS	Fits linear rise in EPSC data from Gittis et al. (2010)
g_{nmda}	0.2nS	Fixed by maintaining the 2:1 AMPA:NMDA ratio from Moyer et al. (2007)
g_{gaba} MSN–MSN	0.75nS	Koos et al. (2004)
g_{gaba} FSI–MSN	3.75nS	Tomkins et al. (2014)
g_{gaba} FSI–FSI	1.1nS	Gittis et al. (2010)
$g_{\text{fs-gap}}$	5ns	Fitted to Galarreta and Hestrin (1999)
β_1	0.5	Tomkins et al. (2014)
β_2	0.3	
ϕ_1, ϕ_2	0.3	
$N_{\text{ampa}}, N_{\text{gaba}}$	2000	Tuning (see text)
N_{nmda}	600	
r_{max}	2000	

Table S10. Striatal microcircuit: Neuropeptide properties

Striatal microcircuit: Neuropeptide properties		
**Parameter**	**Value**	**Source**
β_{sp}	0.47	Blomeley and Bracci (2008)
τ_r^{sp}	10ms	Tuning (see text)
τ_f^{sp}	200ms	
τ_d^{sp}	40ms	
λ_{sp}	5.5	
b_{sp}	2.5	
β_{enk}	0.3	Blomeley and Bracci (2011)
τ_r^{enk}	15ms	Tuning (see text)
τ_f^{enk}	300ms	
τ_d^{enk}	400ms	
λ_{enk}	4.5	
b_{enk}	1	

Table S11. Basal ganglia–thalamocortical loop: Summary

**Basal ganglia loop: Model summary**	
**Populations**	Five: MCtx, STN, GPe, GPi/SNr, VLT
**Topology**	—
**Connectivity**	Within BG loop: One–to–one and all–to–all<br>To striatal microcircuit: Fixed channel–divergent
**Neuron model**	Leaky integrator
**Channel models**	—
**Synapse model**	—
**Plasticity**	—
**Input**	Rate–converted Poisson spike sources representing sensory cortex project to MCtx and STN<br>Rate–converted spiking MSN output from striatal microcircuit projects to GPe and GPi/SNr
**Measurements**	Activity rate output

Table S12. Basal ganglia–thalamocortical loop: Populations

**Basal ganglia loop: Populations**			
**Name**	**Type**	**Size**	**Organisation**
MCtx (mc)<br>STN (stn)<br>GPe (gp)<br>GPi/SNr (snr)<br>VLT (vlt)	Leaky integrator	6	Six action channels $c_1 \dots c_6$ of 1 neuron each

Table S13. Basal ganglia–thalamocortical loop: Connectivity

**Basal ganglia loop: Connectivity**	
**Connection**	**Type**
MCtx $\rightarrow$ VLT<br>MCtx $\rightarrow$ STN	One–to–one
STN $\rightarrow$ GPe<br>STN $\rightarrow$ GPi/SNr	All–to–all
GPe $\rightarrow$ STN<br>GPe $\rightarrow$ GPi/SNr<br>GPi/SNr $\rightarrow$ VLT<br>VLT $\rightarrow$ MCtx	One–to–one

Table S14. Basal ganglia–thalamocortical loop: Neuron models

Basal ganglia loop: Neuron models	
**Activation**	$\dot{a} = k(a - u) + u$
**Output**	$y(t) = F(a(t), \theta) = \begin{cases} 0 & \text{if } a(t) \leq \theta \\ a(t) - \theta & \text{if } \theta < a(t) < 1 - \theta \\ 1 & \text{if } a(t) \geq 1 - \theta \end{cases}$
**Spike–to–rate**	$r_i^s(t) = \sum_i S_s \left[\exp\left(\frac{-(t - t_i)}{\tau_f}\right) - \exp\left(\frac{-(t - t_i)}{\tau_r}\right)\right]$<br>$y_i^s(t) = 1 - \exp\left(-\frac{r_s(t)}{\lambda_s}\right)^{b_s}$<br>Where s is d1, d2 or sc and S_s is the number of spikes arriving from population s
**Name**	**Dynamics**
MCtx	$u_i^{\text{mc}} = w_{\text{mc}} y_i^{\text{sc}} + w_{\text{vlt}} y_i^{\text{vlt}}$<br>$y_i^{\text{mc}} = F\left(a_i^{\text{mc}}, 0\right)$
STN	$u_i^{\text{stn}} = w_{\text{stn}} y_i^{\text{sc}} + w_{\text{stn}} y_i^{\text{mc}} + w_{\text{gp}} y_i^{\text{gp}}$<br>$y_i^{\text{stn}} = F\left(a_i^{\text{stn}}, -0.25\right)$
GPe	$u_i^{\text{gp}} = w_{\text{gp}} \sum_j^n y_j^{\text{stn}} - y_i^{\text{d2}}$<br>$y_i^{\text{gp}} = F\left(a_i^{\text{gp}}, -0.2\right)$
GPi/SNr	$u_i^{\text{snr}} = w_{\text{snr}} \sum_j^n y_j^{\text{stn}} - y_i^{\text{d1}} - w_{\text{gp}} y_i^{\text{gp}}$<br>$y_i^{\text{snr}} = F\left(a_i^{\text{snr}}, -0.2\right)$
VLT	$u_i^{\text{vlt}} = w_{\text{mc}} y_i^{\text{mc}} + w_{\text{snr}} y_i^{\text{snr}}$<br>$y_i^{\text{vlt}} = F\left(a_i^{\text{vlt}}, 0\right)$

Table S15. Basal ganglia–thalamocortical loop: Inputs

Basal ganglia loop: Inputs		
**Input**	**Targets**	
	STN	MCtx
Sensory cortex (sc)	$\text{sc}_c^i \rightarrow \text{stn}_c, 1 \leq i \leq 500$	$\text{sc}_c^i \rightarrow \text{mc}_c, 1 \leq i \leq 500$
	GPi/SNr	GPe
D1 / D2 MSNs (d1 / d2)	$\text{d1}_c^i \rightarrow \text{snr}_c, 1 \leq i \leq 500$	$\text{d2}_c^i \rightarrow \text{gp}_c, 1 \leq i \leq 500$

Table S16. Basal ganglia–thalamocortical loop: Weights and properties

Basal ganglia loop: Weights and properties		
**Parameter**	**Value**	**Source**
$w_{\text{sc–d1}}$	0.5	Humphries and Gurney (2002)
$w_{\text{sc–d2}}$	0.5	
$w_{\text{sc–stn}}$	0.5	
$w_{\text{sc–mc}}$	0.5	Tuning (see text)
$w_{\text{mc–d1}}$	0.5	Humphries and Gurney (2002)
$w_{\text{mc–d2}}$	0.5	
$w_{\text{mc–stn}}$	0.5	
$w_{\text{mc–vlt}}$	1	
$w_{\text{vlt–mc}}$	1.05	Tuning (see text)
$w_{\text{d1–snr}}$	-1	Humphries and Gurney (2002)
$w_{\text{d2–gp}}$	-1	
$w_{\text{stn–snr}}$	0.8	
$w_{\text{stn–gp}}$	0.8	
$w_{\text{gp–stn}}$	-1	
$w_{\text{gp–snr}}$	-0.4	
$w_{\text{snr–vlt}}$	-1	
k	0.9608	
τ_r	9ms	Tuning (See text)
τ_f	10ms	
$\lambda_{\text{d1}}, \lambda_{\text{d2}}$	15	
λ_{sc}	850	
$b_{\text{d1}}, b_{\text{d2}}$	1	
b_{sc}	1.5	

REFERENCES

Blomeley, C. and Bracci, E. (2008). Substance P depolarizes striatal projection neurons and facilitates their glutamatergic inputs. *The Journal of Physiology* 586, 2143–2155. doi:10.1113/jphysiol.2007.148965

Blomeley, C. P. and Bracci, E. (2011). Opioidergic Interactions between Striatal Projection Neurons. *Journal of Neuroscience* 31, 13346–13356. doi:10.1523/JNEUROSCI.1775-11.2011

Bracci, E., Centonze, D., Bernardi, G., and Calabresi, P. (2002). Dopamine Excites Fast-Spiking Interneurons in the Striatum. *Journal of Neurophysiology* 87, 2190–2194. doi:10.1152/jn.00754.2001

Galarreta, M. and Hestrin, S. (1999). A network of fast-spiking cells in the neocortex connected by electrical synapses. *Nature* 402, 72–75. doi:10.1038/47029

Gittis, A. H., Nelson, A. B., Thwin, M. T., Palop, J. J., and Kreitzer, A. C. (2010). Distinct Roles of GABAergic Interneurons in the Regulation of Striatal Output Pathways. *The Journal of Neuroscience* 30, 2223–2234. doi:10.1523/JNEUROSCI.4870-09.2010

Gorelova, N., Seamans, J. K., and Yang, C. R. (2002). Mechanisms of Dopamine Activation of Fast-Spiking Interneurons That Exert Inhibition in Rat Prefrontal Cortex. *Journal of Neurophysiology* 88, 3150–3166. doi:10.1152/jn.00335.2002

Humphries, M. and Gurney, K. (2002). The role of intra-thalamic and thalamocortical circuits in action selection. *Network: Computation in Neural Systems* 13, 131–156. doi:10.1080/net.13.1.131.156

Humphries, M. D., Lepora, N., Wood, R., and Gurney, K. (2009). Capturing dopaminergic modulation and bimodal membrane behaviour of striatal medium spiny neurons in accurate, reduced models. *Frontiers in Computational Neuroscience* 3. doi:10.3389/neuro.10.026.2009

Humphries, M. D., Wood, R., and Gurney, K. (2010). Reconstructing the Three-Dimensional GABAergic Microcircuit of the Striatum. *PLoS Computational Biology* 6, e1001011. doi:10.1371/journal.pcbi.1001011

Izhikevich, E. M. (2007). *Dynamical Systems in Neuroscience* (MIT Press)

Jahr, C. E. and Stevens, C. F. (1990). A quantitative description of NMDA receptor-channel kinetic behavior. *The Journal of Neuroscience* 10, 1830–1837

Koos, T., Tepper, J. M., and Wilson, C. J. (2004). Comparison of IPSCs Evoked by Spiny and Fast-Spiking Neurons in the Neostriatum. *The Journal of Neuroscience* 24, 7916–7922. doi:10.1523/JNEUROSCI.2163-04.2004

Mahon, S., Deniau, J.-M., Charpier, S., and Delord, B. (2000). Role of a Striatal Slowly Inactivating Potassium Current in Short-Term Facilitation of Corticostriatal Inputs: A Computer Simulation Study. *Learning & Memory* 7, 357–362. doi:10.1101/lm.34800

Moyer, J. T., Wolf, J. A., and Finkel, L. H. (2007). Effects of Dopaminergic Modulation on the Integrative Properties of the Ventral Striatal Medium Spiny Neuron. *Journal of Neurophysiology* 98, 3731–3748. doi:10.1152/jn.00335.2007

Nordlie, E., Gewaltig, M.-O., and Plesser, H. E. (2009). Towards Reproducible Descriptions of Neuronal Network Models. *PLOS Comput Biol* 5, e1000456. doi:10.1371/journal.pcbi.1000456

Tateno, T., Harsch, A., and Robinson, H. P. C. (2004). Threshold Firing Frequency–Current Relationships of Neurons in Rat Somatosensory Cortex: Type 1 and Type 2 Dynamics. *Journal of Neurophysiology* 92, 2283–2294. doi:10.1152/jn.00109.2004

Tomkins, A., Vasilaki, E., Beste, C., Gurney, K., and Humphries, M. D. (2014). Transient and steady-state selection in the striatal microcircuit. *Frontiers in Computational Neuroscience* 7. doi:10.3389/fncom.2013.00192
