# Supplementary figures and images for: Striatal Neuropeptides Enhance Selection and Rejection of Sequential Actions
Source: Front Comput Neurosci. 2017 Jul 27;11:62. doi: 10.3389/fncom.2017.00062 (PMC5529366; doi:10.3389/fncom.2017.00062)

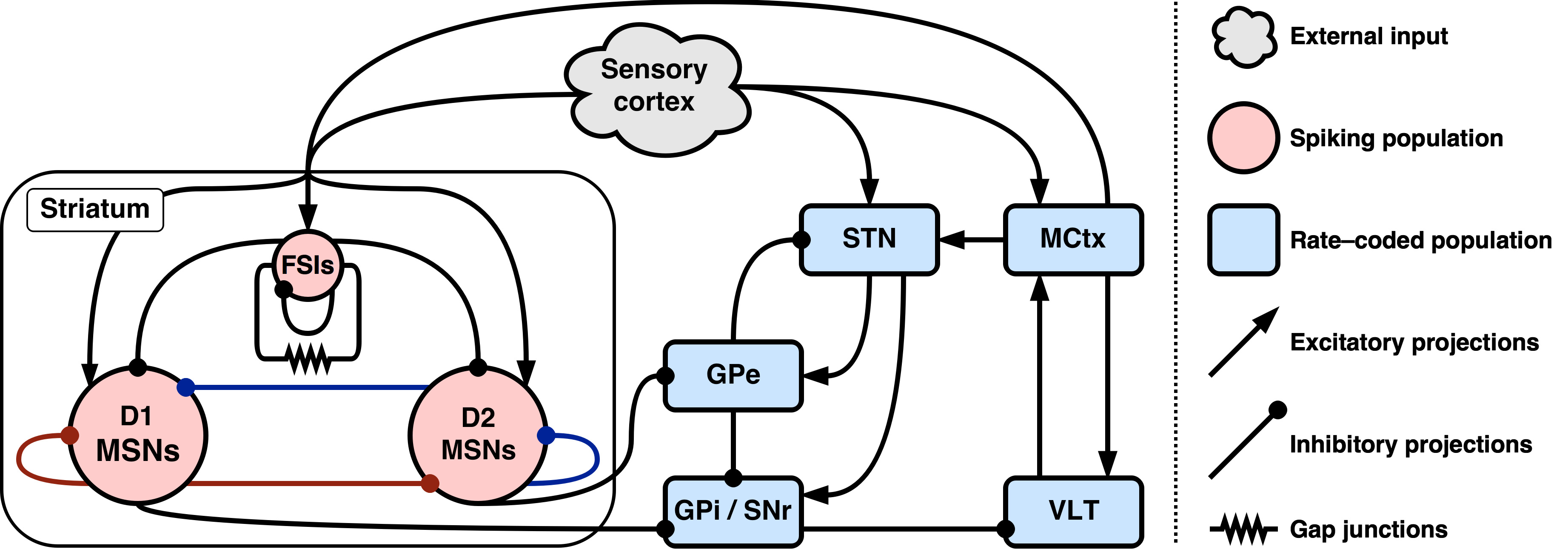

Supplement: Supplementary file 2 [file Presentation1.ZIP › 01_model_diagram.tiff]

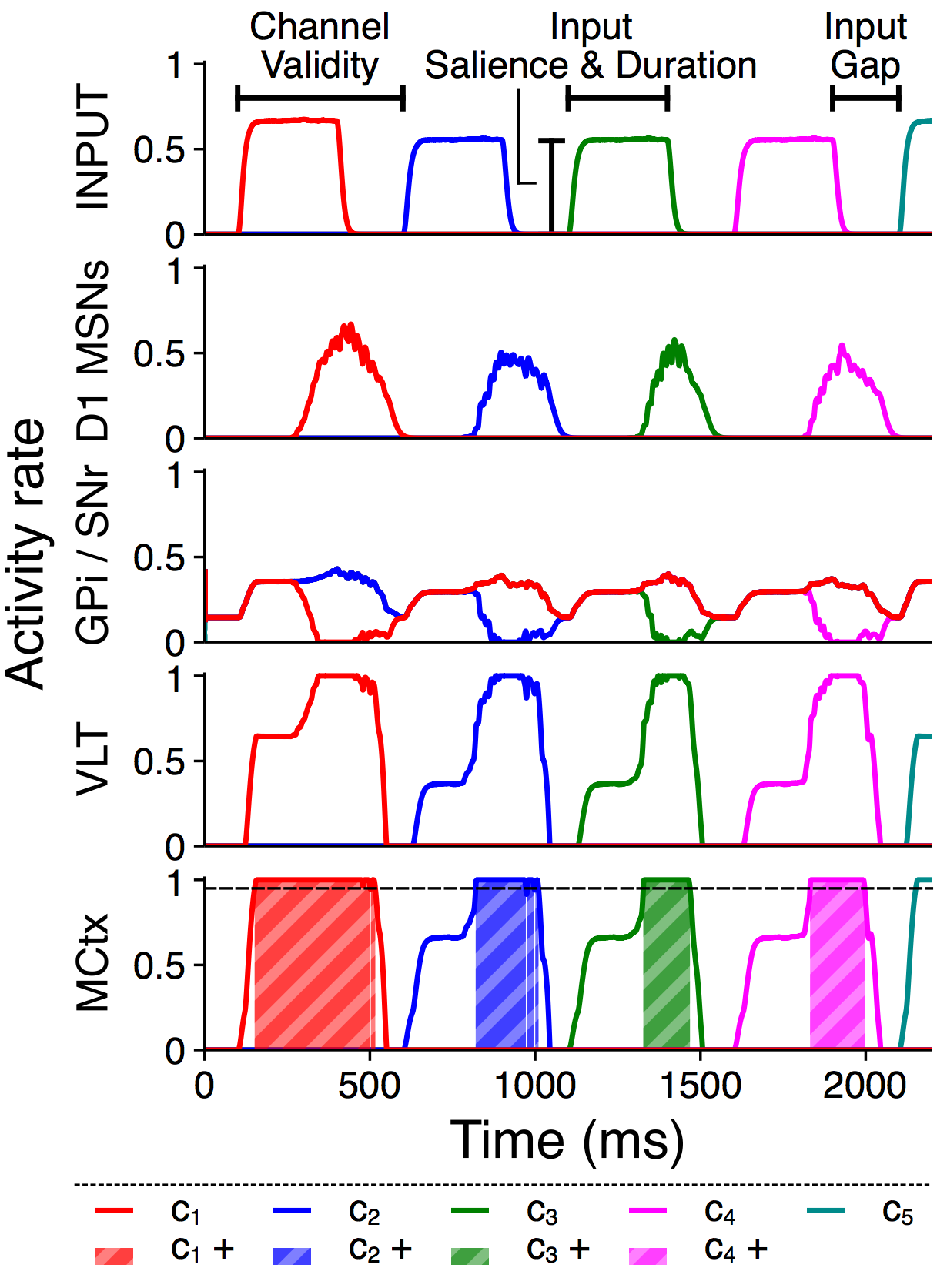

Supplement: Supplementary file 2 [file Presentation1.ZIP › 02_seq_ctrl_300-1600.tiff]

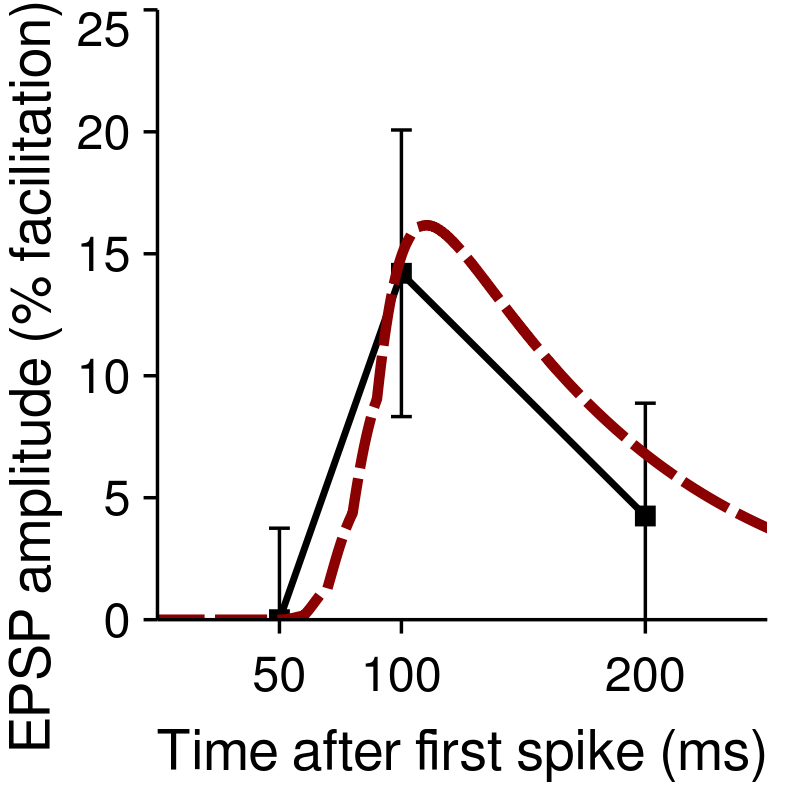

Supplement: Supplementary file 2 [file Presentation1.ZIP › 03-a_sp_5spike.tiff]

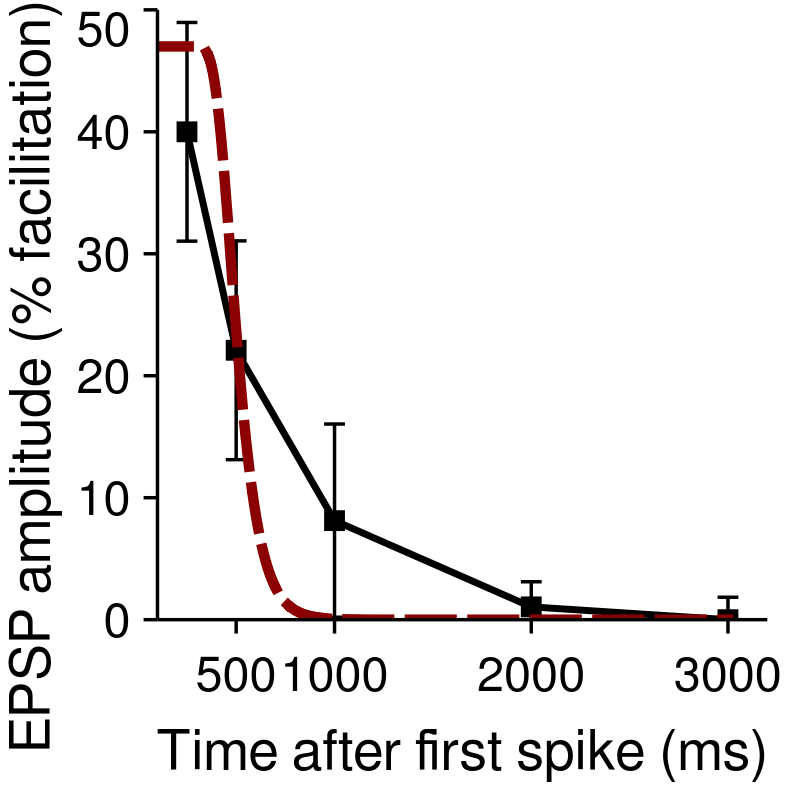

Supplement: Supplementary file 2 [file Presentation1.ZIP › 03-b_sp_antidromic.tiff]

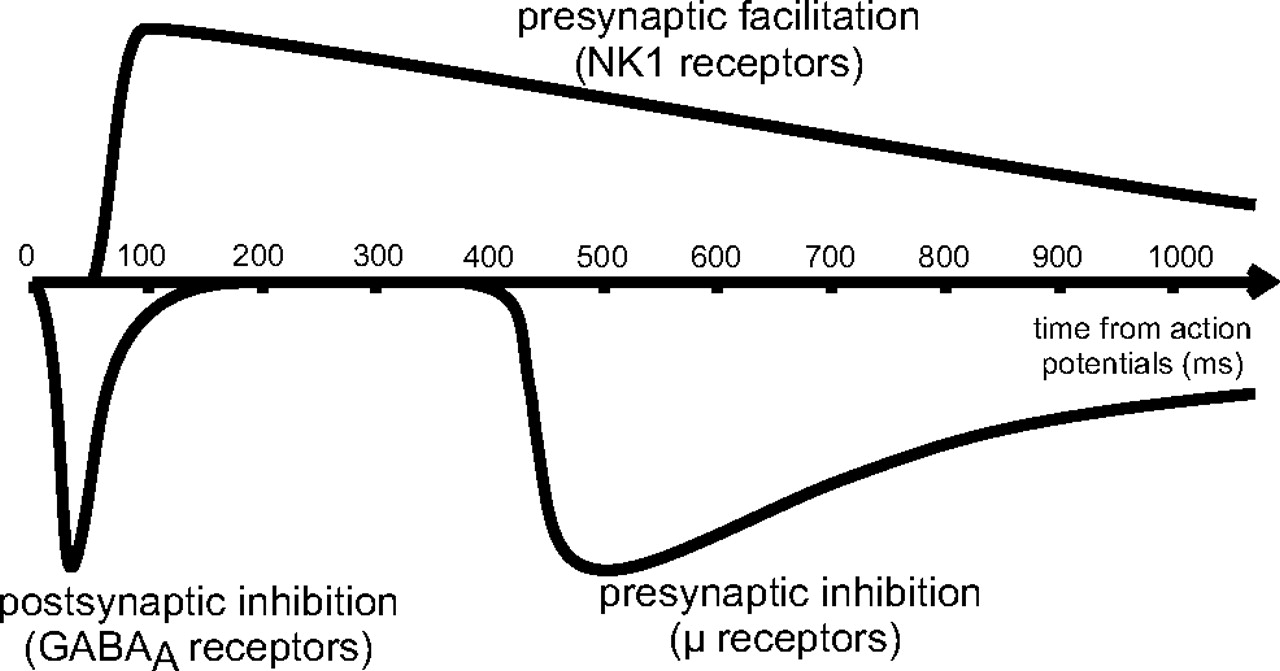

Supplement: Supplementary file 2 [file Presentation1.ZIP › 03-c_np_curve_1.jpg]

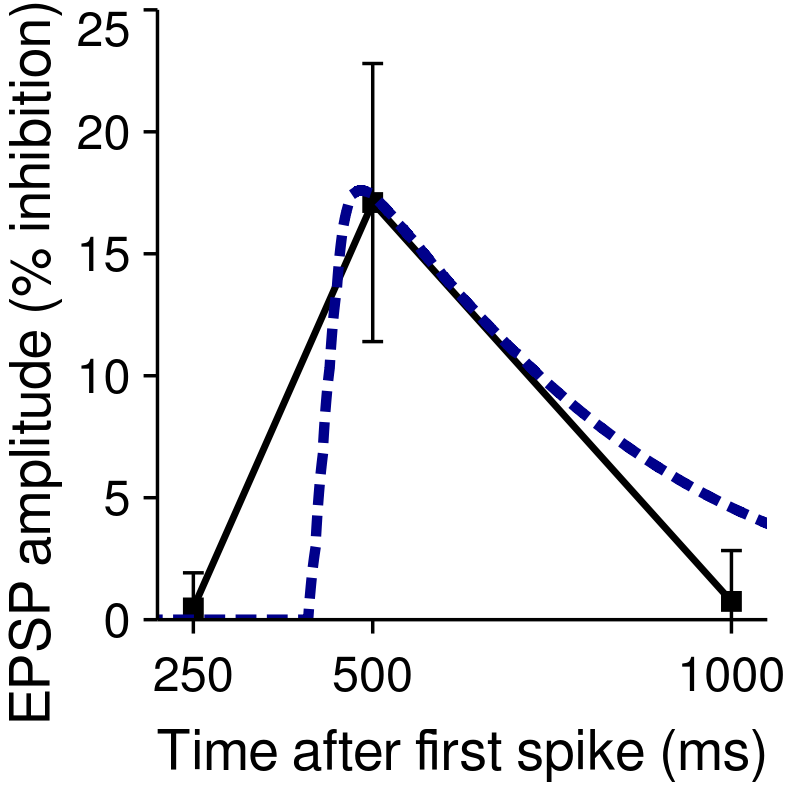

Supplement: Supplementary file 2 [file Presentation1.ZIP › 03-d_enk_5spike.tiff]

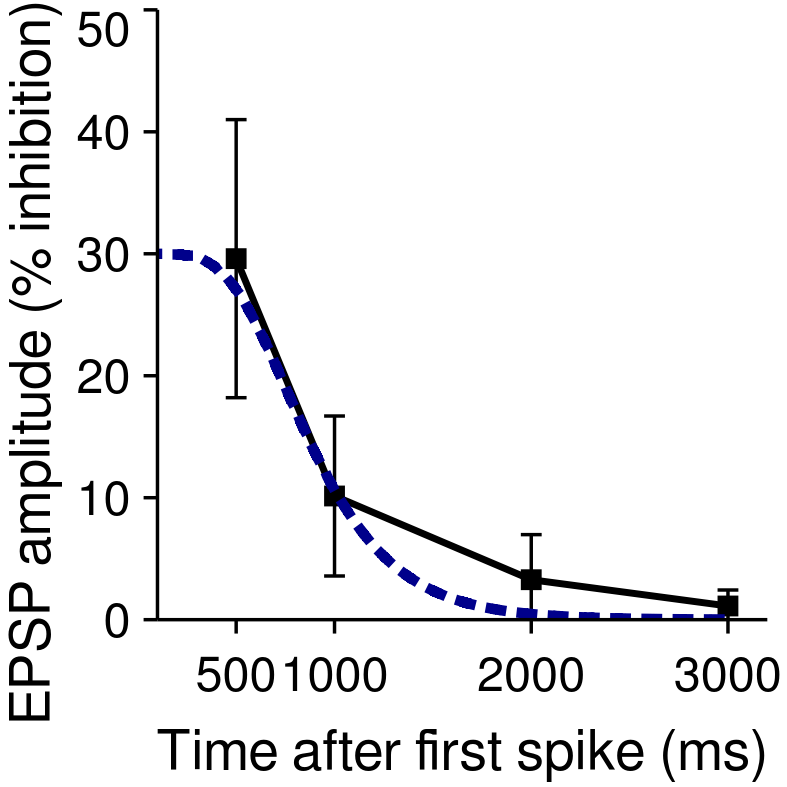

Supplement: Supplementary file 2 [file Presentation1.ZIP › 03-e_enk_antidromic.tiff]

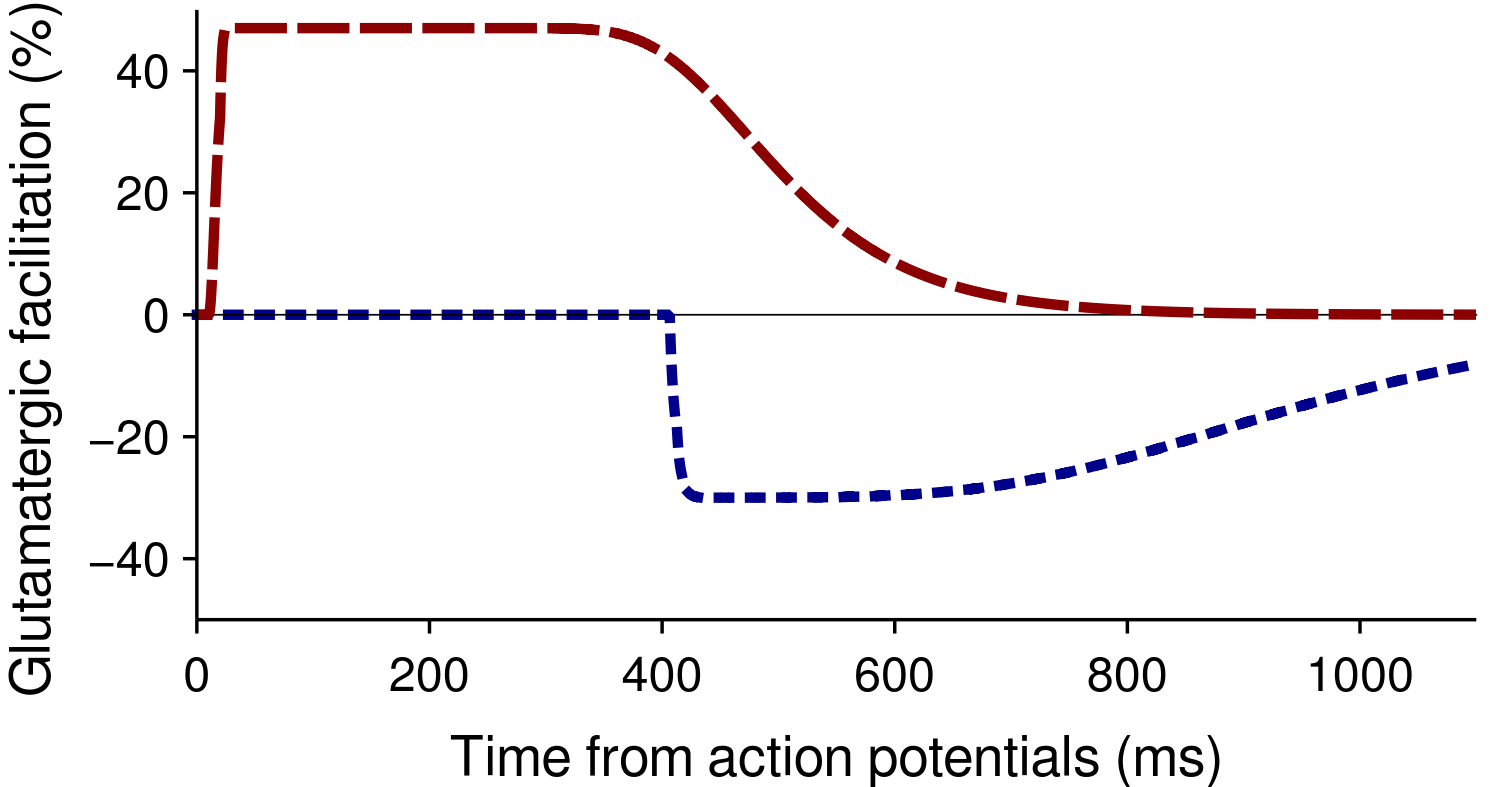

Supplement: Supplementary file 2 [file Presentation1.ZIP › 03-f_np_curve_2.tiff]

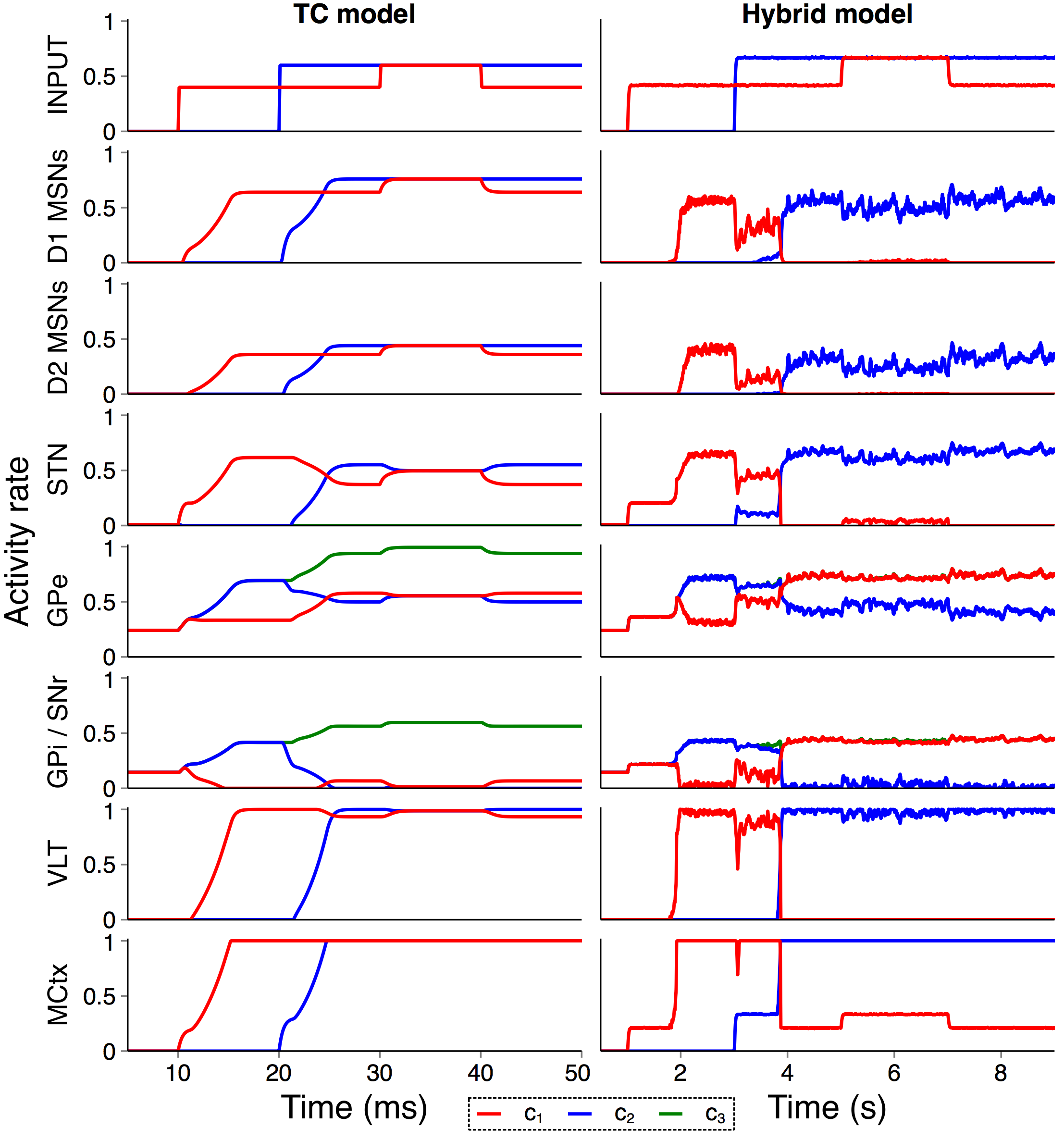

Supplement: Supplementary file 2 [file Presentation1.ZIP › 04_test_comparison.tiff]

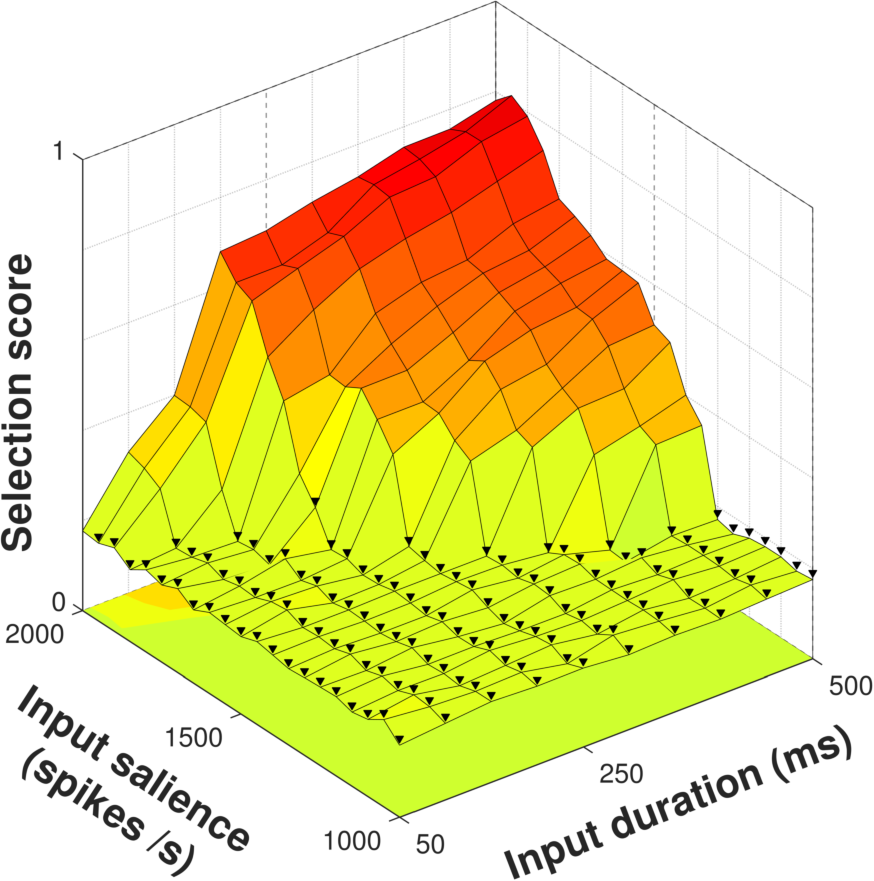

Supplement: Supplementary file 2 [file Presentation1.ZIP › 05-a_series_ctrl.tiff]

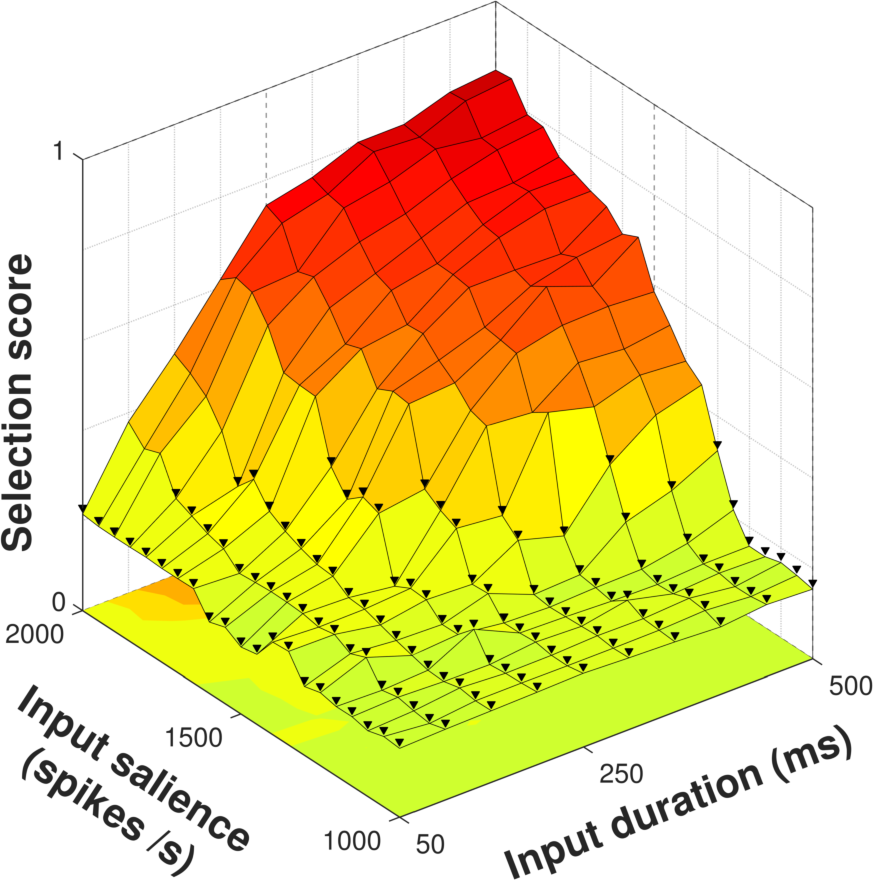

Supplement: Supplementary file 2 [file Presentation1.ZIP › 05-b_series_diffuse.tiff]

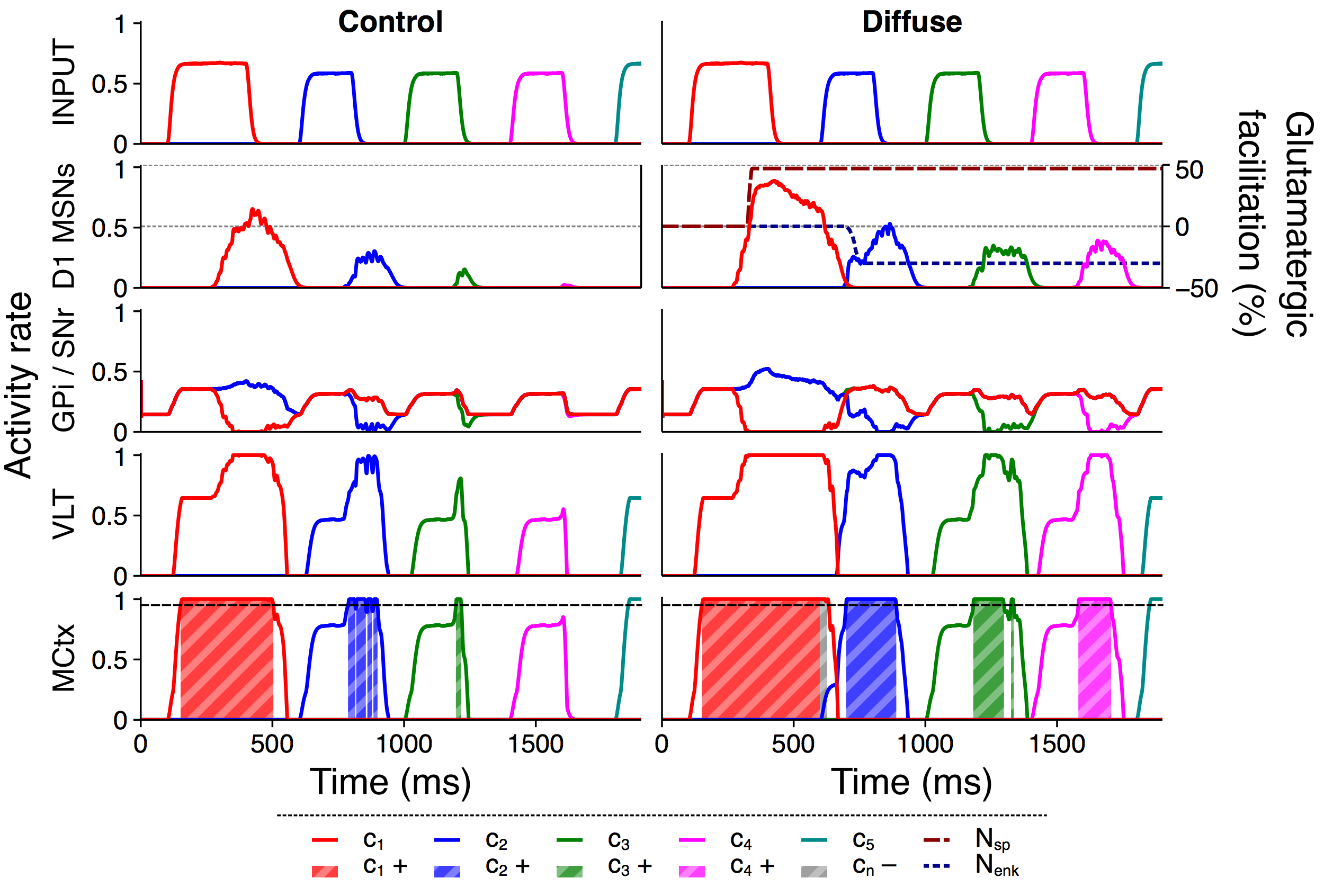

Supplement: Supplementary file 2 [file Presentation1.ZIP › 06_series_200-1700_comparison.tiff]

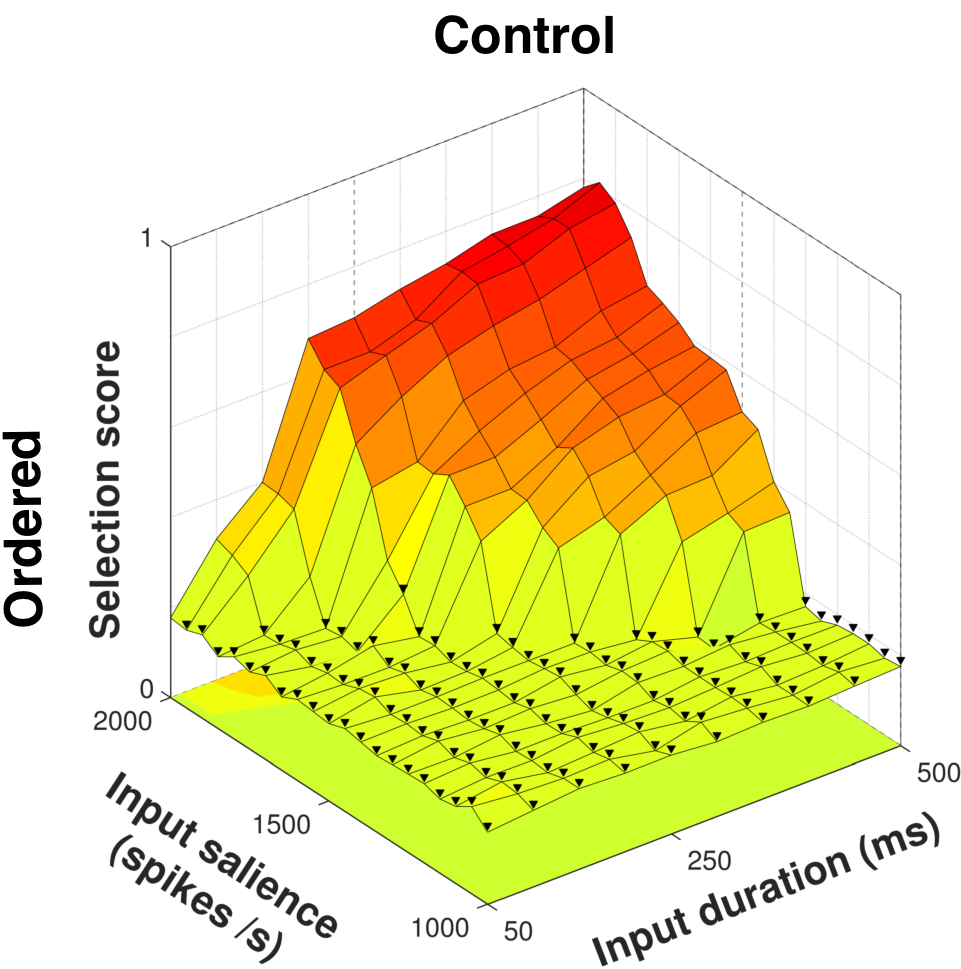

Supplement: Supplementary file 2 [file Presentation1.ZIP › 07-a_seq_ctrl.tiff]

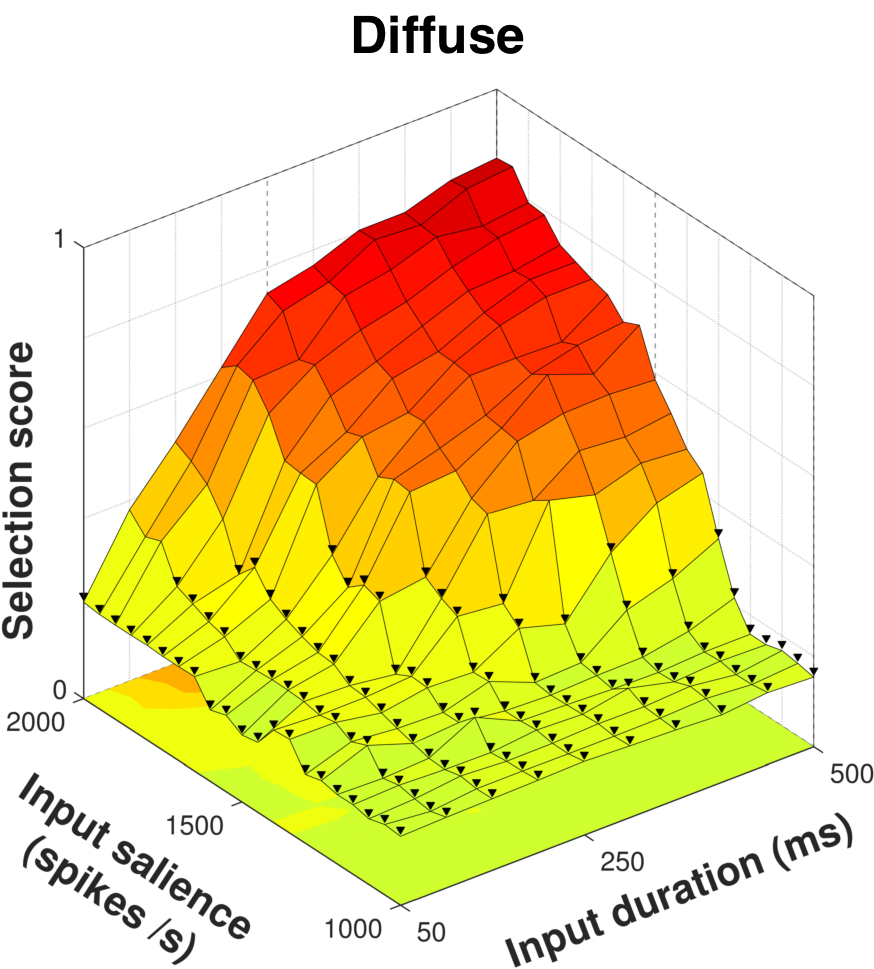

Supplement: Supplementary file 2 [file Presentation1.ZIP › 07-b_seq_diffuse.tiff]

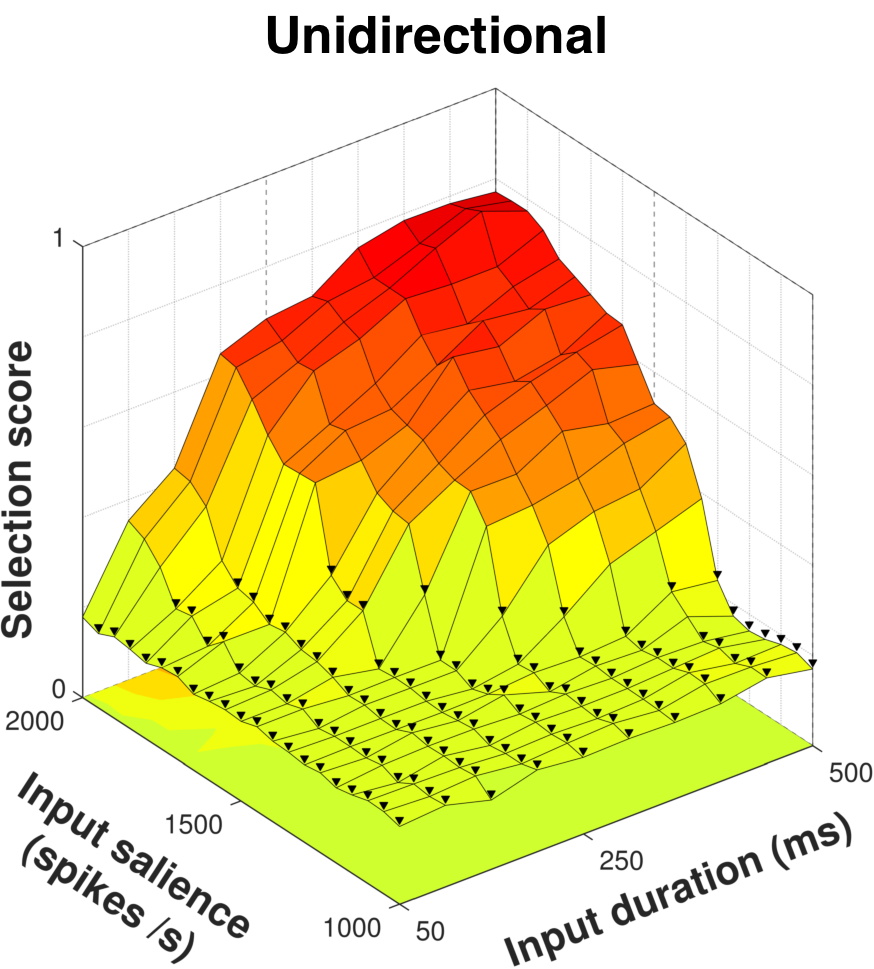

Supplement: Supplementary file 2 [file Presentation1.ZIP › 07-c_seq_uni.tiff]

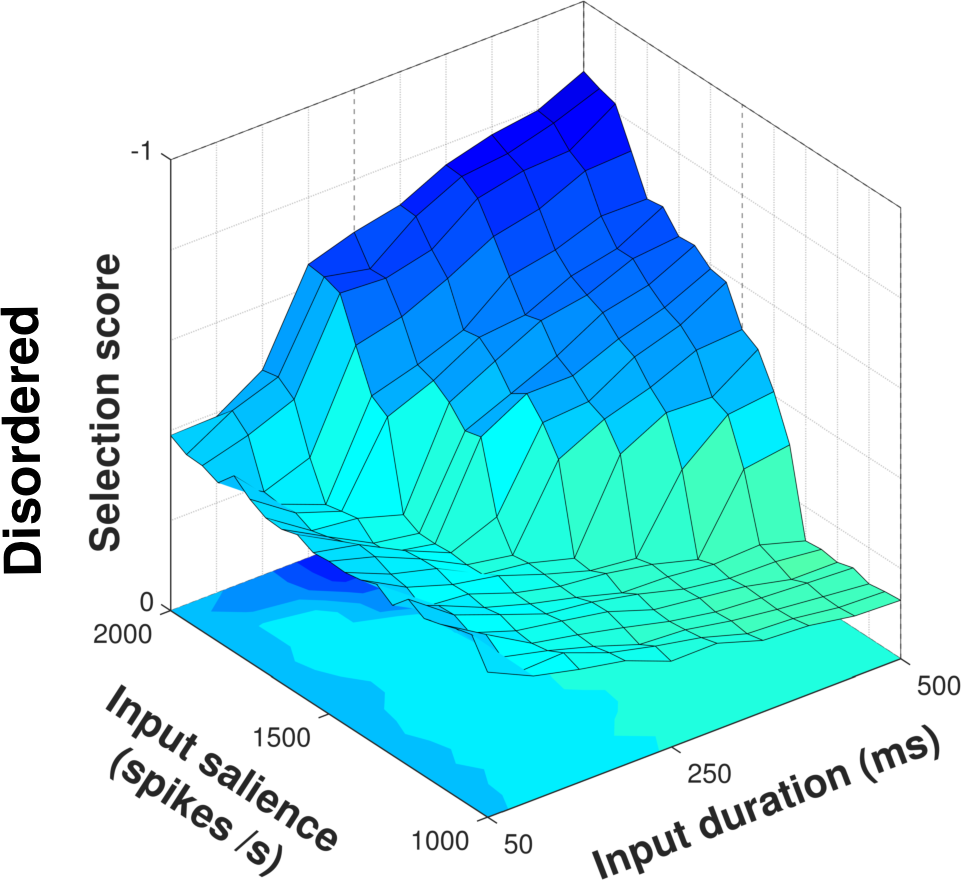

Supplement: Supplementary file 2 [file Presentation1.ZIP › 07-d_rev_ctrl.tiff]

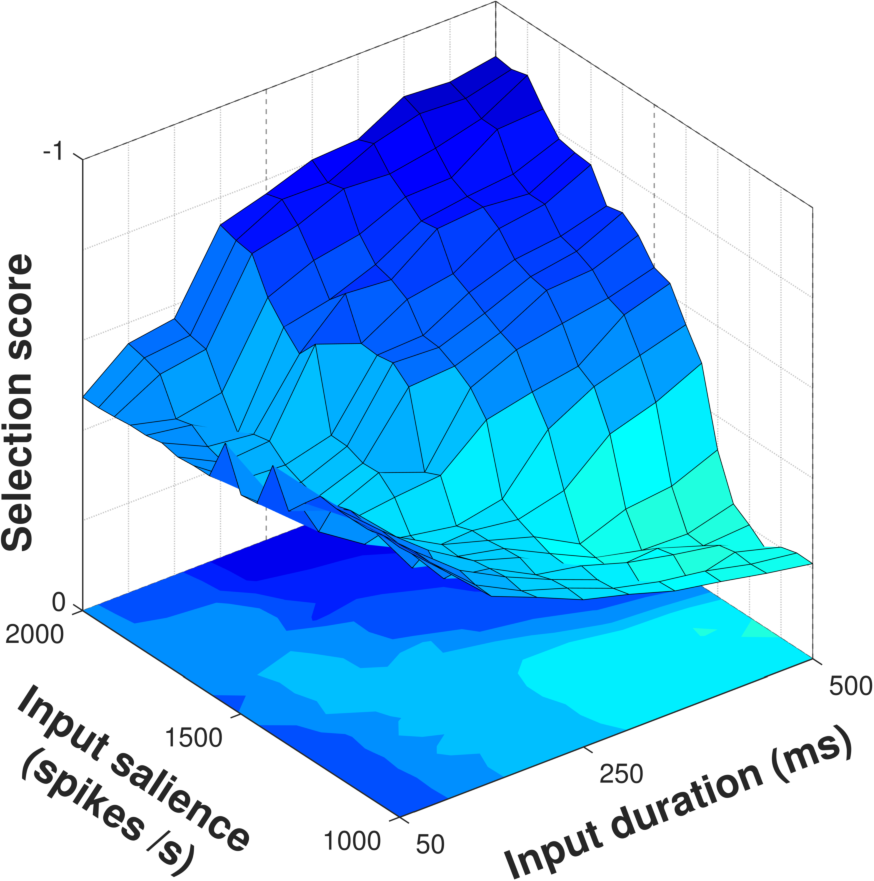

Supplement: Supplementary file 2 [file Presentation1.ZIP › 07-e_rev_diffuse.tiff]

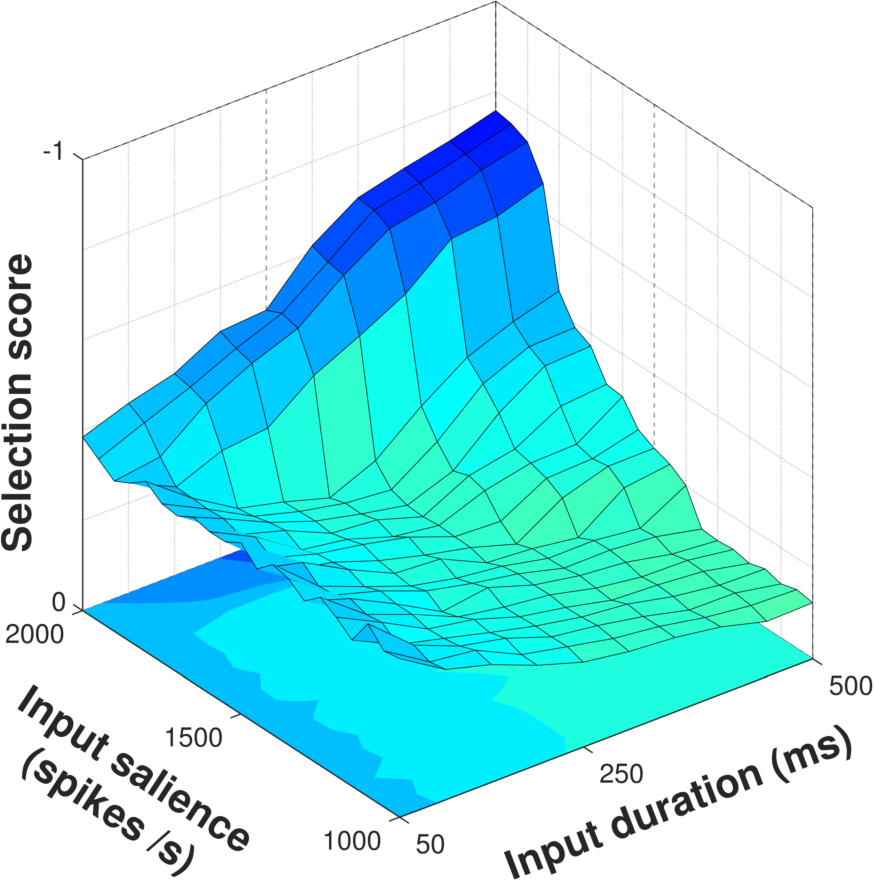

Supplement: Supplementary file 2 [file Presentation1.ZIP › 07-f_rev_uni.tiff]

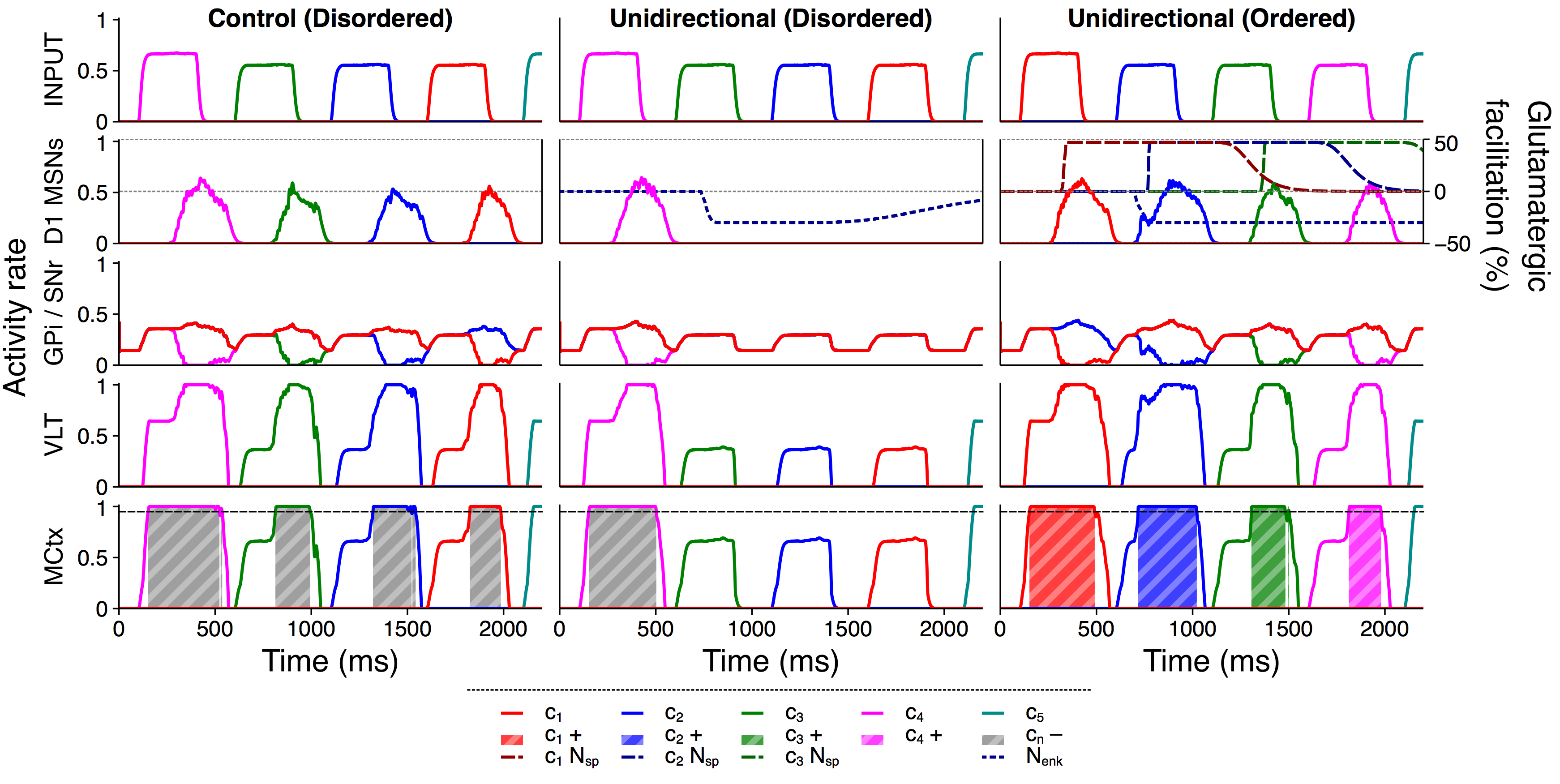

Supplement: Supplementary file 2 [file Presentation1.ZIP › 08_rev_300-1600_comparison.tiff]

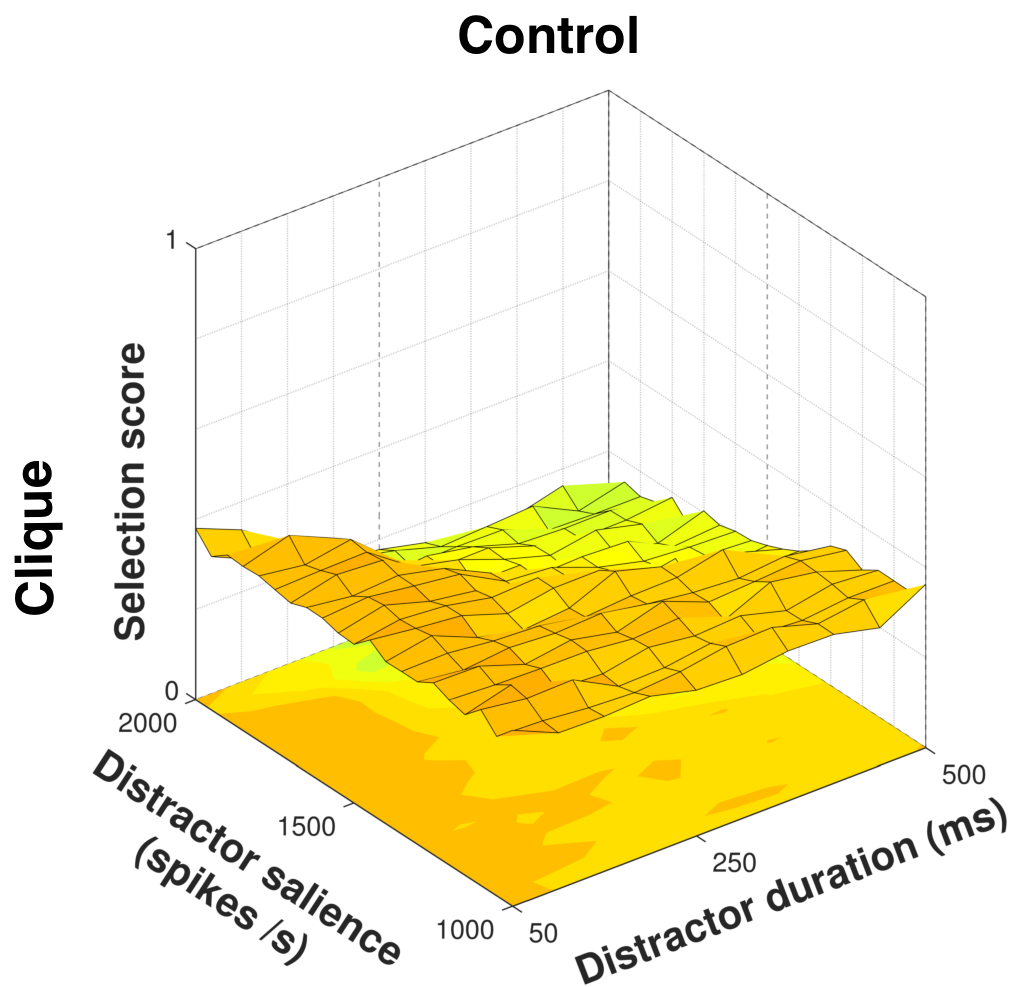

Supplement: Supplementary file 2 [file Presentation1.ZIP › 09-a_clique_ctrl_sequence.tiff]

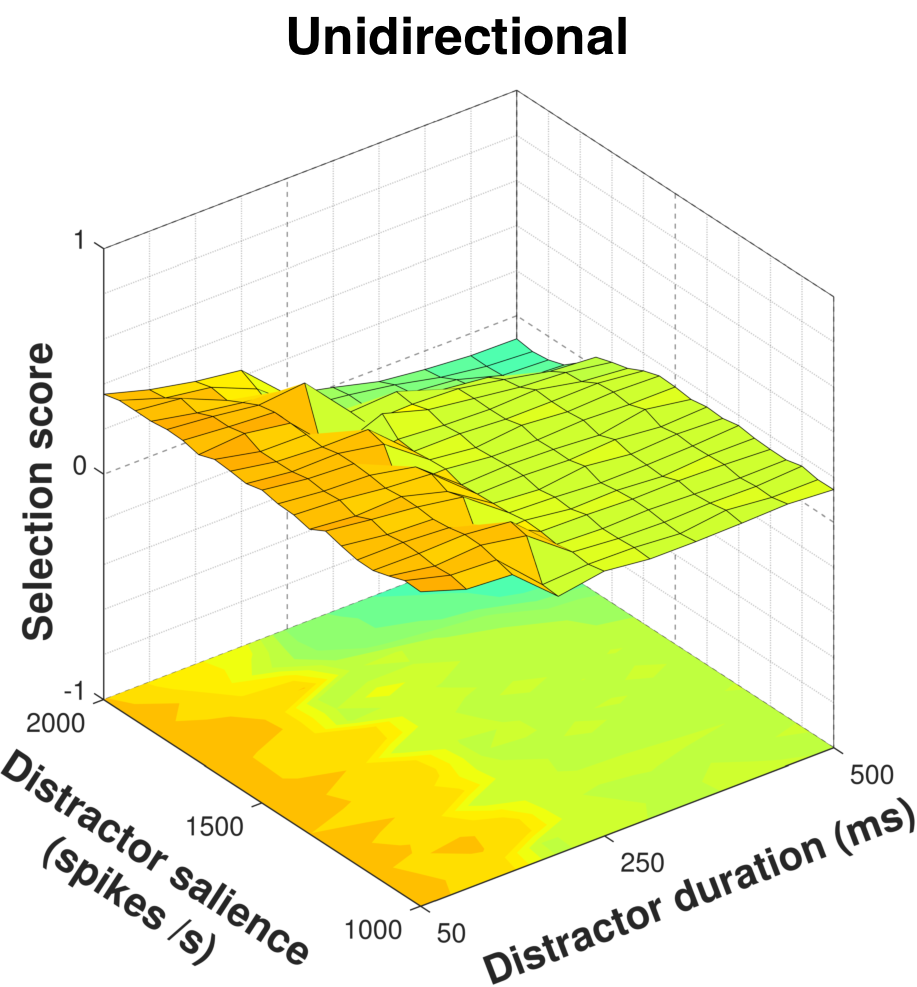

Supplement: Supplementary file 2 [file Presentation1.ZIP › 09-b_clique_uni_sequence.tiff]

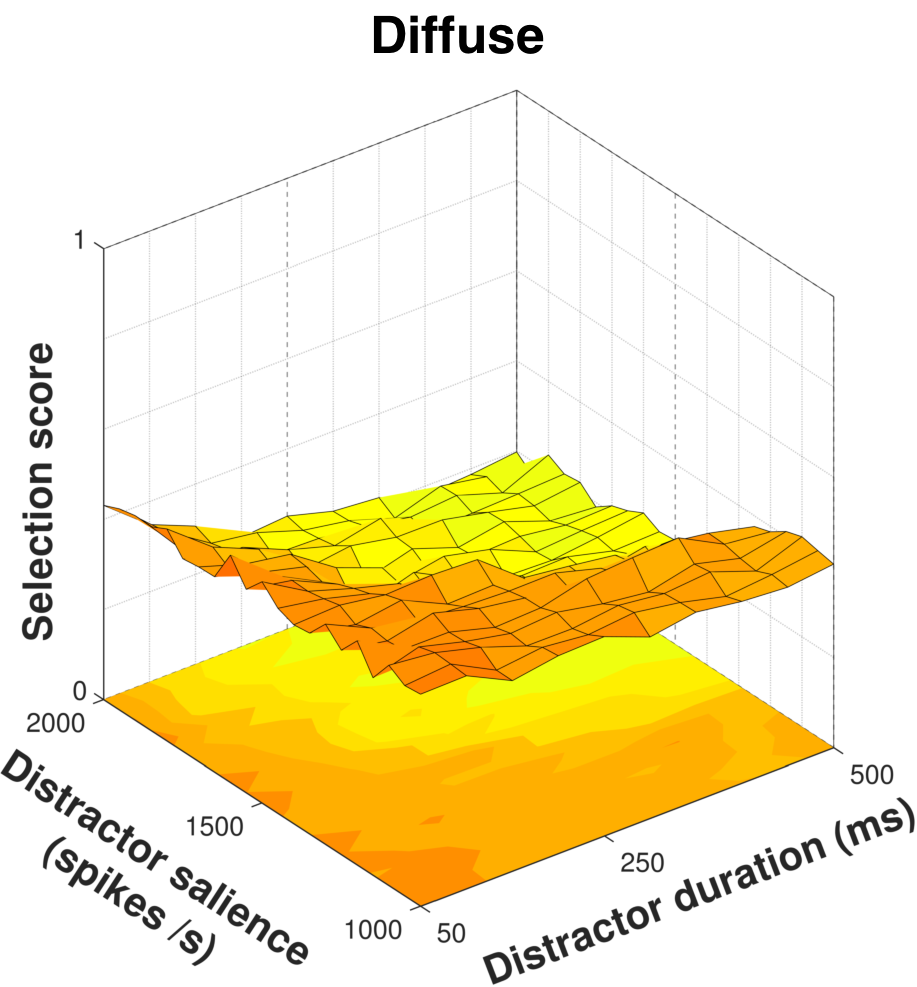

Supplement: Supplementary file 2 [file Presentation1.ZIP › 09-c_clique_diffuse_sequence.tiff]

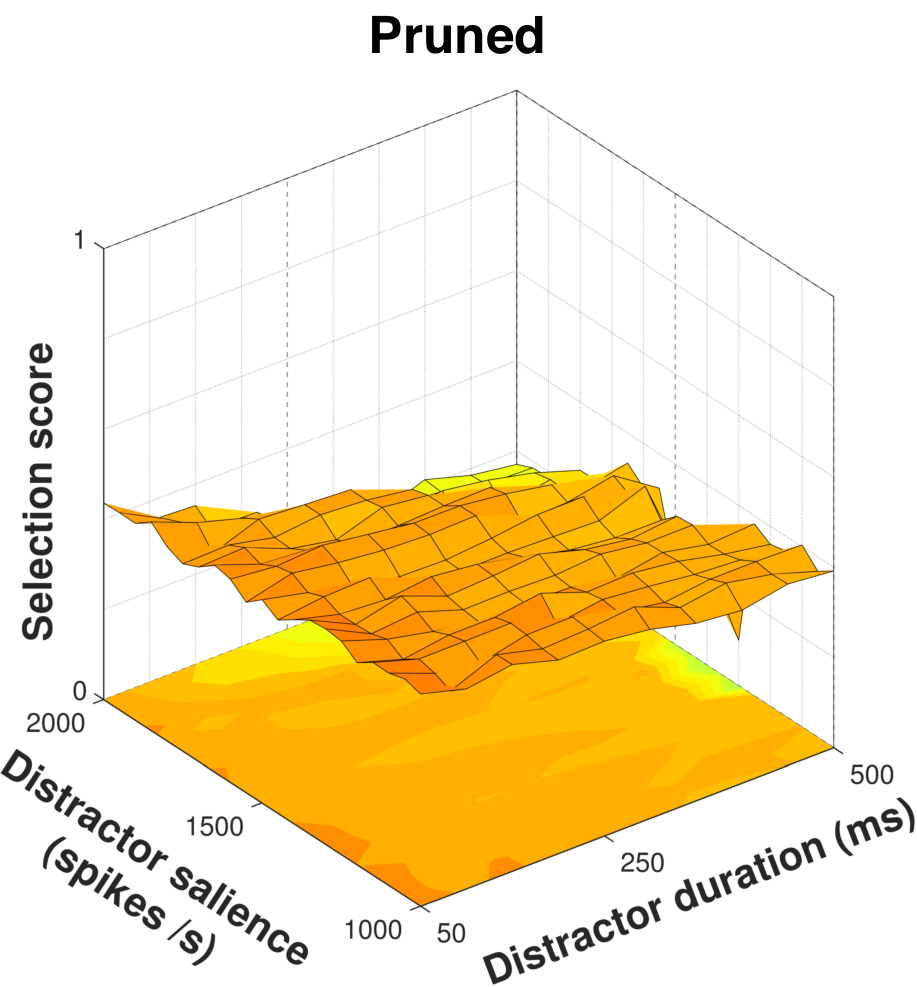

Supplement: Supplementary file 2 [file Presentation1.ZIP › 09-d_clique_pruned_sequence.tiff]

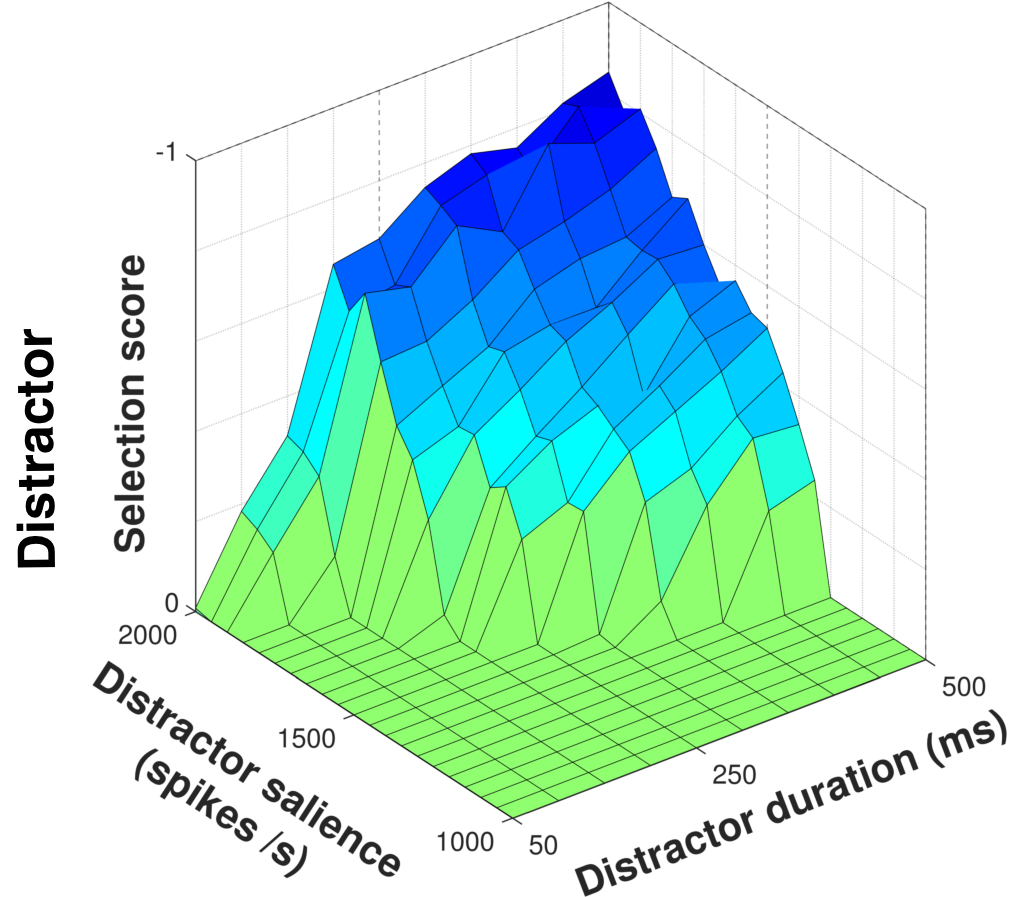

Supplement: Supplementary file 2 [file Presentation1.ZIP › 09-e_clique_ctrl_distractor.tiff]

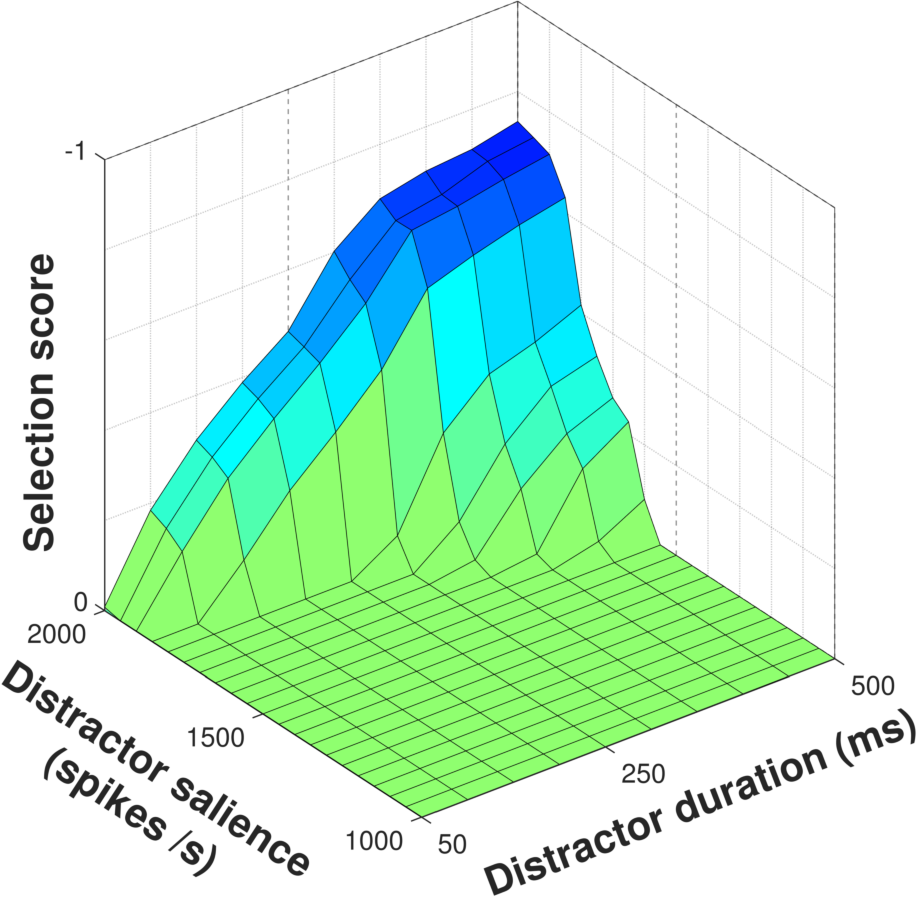

Supplement: Supplementary file 2 [file Presentation1.ZIP › 09-f_clique_uni_distractor.tiff]

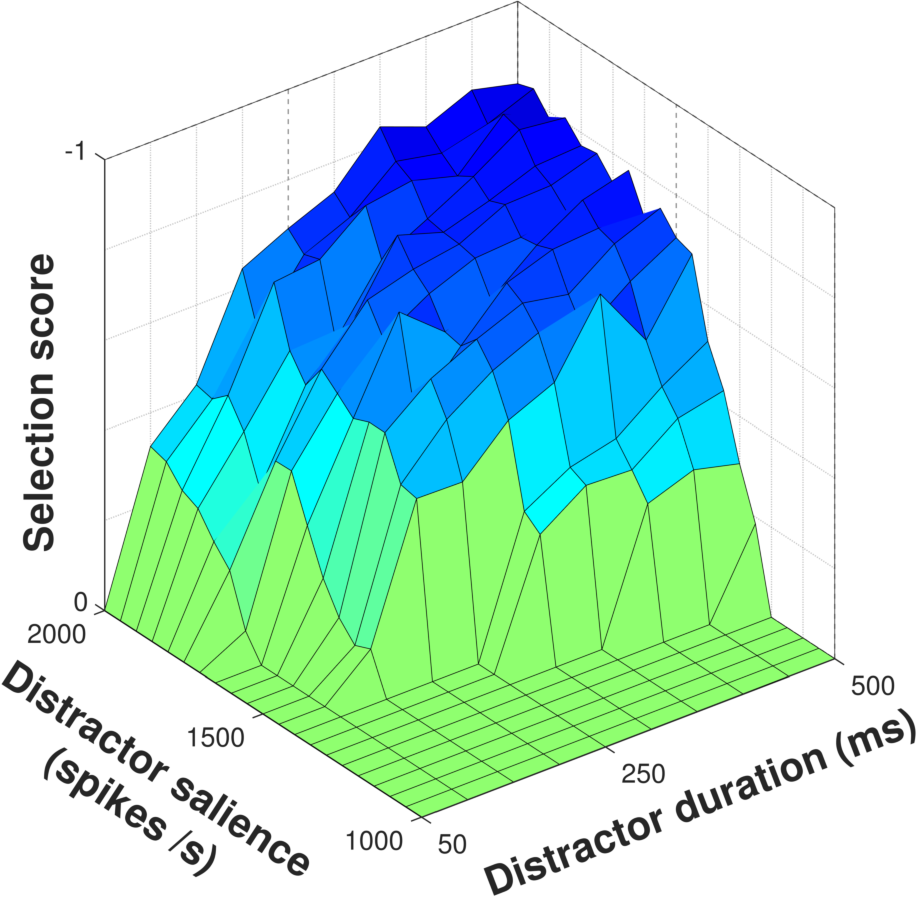

Supplement: Supplementary file 2 [file Presentation1.ZIP › 09-g_clique_diffuse_distractor.tiff]

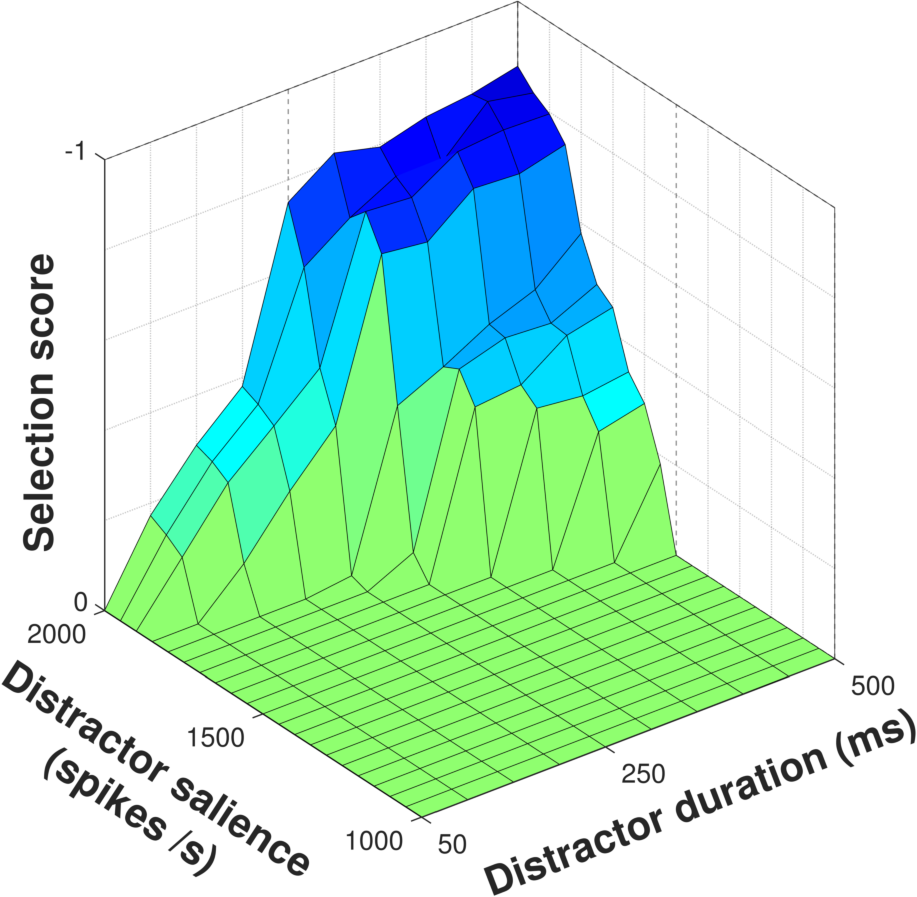

Supplement: Supplementary file 2 [file Presentation1.ZIP › 09-h_clique_pruned_distractor.tiff]
